# The Wild Sugarcane and Sorghum Kinomes: Insights Into Expansion, Diversification, and Expression Patterns

**DOI:** 10.3389/fpls.2021.668623

**Published:** 2021-07-07

**Authors:** Alexandre Hild Aono, Ricardo José Gonzaga Pimenta, Ana Letycia Basso Garcia, Fernando Henrique Correr, Guilherme Kenichi Hosaka, Marishani Marin Carrasco, Cláudio Benício Cardoso-Silva, Melina Cristina Mancini, Danilo Augusto Sforça, Lucas Borges dos Santos, James Shiniti Nagai, Luciana Rossini Pinto, Marcos Guimarães de Andrade Landell, Monalisa Sampaio Carneiro, Thiago Willian Balsalobre, Marcos Gonçalves Quiles, Welison Andrade Pereira, Gabriel Rodrigues Alves Margarido, Anete Pereira de Souza

**Affiliations:** ^1^Center for Molecular Biology and Genetic Engineering (CBMEG), University of Campinas (UNICAMP), Campinas, Brazil; ^2^Department of Genetics, Luiz de Queiroz College of Agriculture (ESALQ), University of São Paulo (USP), Piracicaba, Brazil; ^3^Faculty of Medicine, Institute for Computational Genomics, RWTH Aachen University, Aachen, Germany; ^4^Advanced Center of Sugarcane Agrobusiness Technological Research, Agronomic Institute of Campinas (IAC), Ribeirão Preto, Brazil; ^5^Departamento de Biotecnologia e Produção Vegetal e Animal, Centro de Ciências Agrárias, Universidade Federal de São Carlos (UFSCar), São Carlos, Brazil; ^6^Instituto de Ciência e Tecnologia (ICT), Universidade Federal de São Paulo (Unifesp), São José dos Campos, Brazil; ^7^Department of Biology, Federal University of Lavras (UFLA), Lavras, Brazil; ^8^Department of Plant Biology, Institute of Biology, University of Campinas (UNICAMP), Campinas, Brazil

**Keywords:** coexpression networks, kinase gene family, phylogenetic analyses, RNA-Seq, *Saccharum spontaneum*, *Sorghum bicolor*

## Abstract

The protein kinase (PK) superfamily is one of the largest superfamilies in plants and the core regulator of cellular signaling. Despite this substantial importance, the kinomes of sugarcane and sorghum have not been profiled. Here, we identified and profiled the complete kinomes of the polyploid *Saccharum spontaneum* (Ssp) and *Sorghum bicolor* (Sbi), a close diploid relative. The Sbi kinome was composed of 1,210 PKs; for Ssp, we identified 2,919 PKs when disregarding duplications and allelic copies, and these were related to 1,345 representative gene models. The Ssp and Sbi PKs were grouped into 20 groups and 120 subfamilies and exhibited high compositional similarities and evolutionary divergences. By utilizing the collinearity between the species, this study offers insights into Sbi and Ssp speciation, PK differentiation and selection. We assessed the PK subfamily expression profiles *via* RNA-Seq and identified significant similarities between Sbi and Ssp. Moreover, coexpression networks allowed inference of a core structure of kinase interactions with specific key elements. This study provides the first categorization of the allelic specificity of a kinome and offers a wide reservoir of molecular and genetic information, thereby enhancing the understanding of Sbi and Ssp PK evolutionary history.

## Introduction

Sugarcane is one of the world’s most important crops, with the highest production quantity and the sixth highest net production value in 2016 (FAO, 2020). For years, this crop has accounted for approximately 80% of the worldwide sugar production (ISO, 2020) and is predicted to account for nearly 40% of the planet’s first-generation biofuel supply in the near future (Lalman et al., 2016). However, it is also known for its unprecedented genomic complexity; modern cultivars arose from interspecific crosses between two autopolyploid species, namely *Saccharum officinarum* (2*n* = 8x = 80, *x* = 10; D’Hont et al., 1998) and the wild *Saccharum spontaneum* (2*n* = 5x =40 to 16x = 128; *x* = 8; Panje and Babu, 1960). These hybridizations produced large (∼10 Gb), highly polyploid and aneuploid genomes (Sforça et al., 2019).

Sugarcane genomic research is hampered by this genomic complexity, and for many years, depended on resources from a closely related species: sorghum (*Sorghum bicolor*). *S. bicolor* (Sbi) is a stress-resistant, multifunctional cereal crop that is primarily grown as a staple food in Africa but can also be used for fodder, sugar, and biofuel production (Serna-Saldívar et al., 2012). *Saccharum* and *Sorghum* belong to the Saccharinae subtribe of the Poaceae family (Clayton, 1987); however, unlike sugarcane, *Sorghum* has not undergone recent polyploidization events (∼96 million years; Guo et al., 2019) and thus has a diploid and much smaller genome that was fully sequenced in 2009 (Paterson et al., 2009). Due to both the evolutionary proximity between the two species and the extensive collinearity between their chromosomes, sorghum has historically been considered a diploid model for sugarcane (Grivet and Arruda, 2002).

The superfamily of protein kinases (PKs) comprises the enzymes responsible for catalyzing the reversible phosphorylation of proteins – one of the most widespread posttranslational modifications across all living organisms. PKs act by transferring the terminal phosphate group from ATP to the hydroxyl group of a serine, threonine, or tyrosine residue in the target protein (Hunter, 1995). These reactions are key events regulating the activity and interactions of proteins; therefore, PKs are relevant in many cellular and metabolic processes (Champion et al., 2004). In plants, they are involved in the regulation of circadian rhythms and cell cycles, the modulation of various developmental and intracellular processes, and the control of cellular cycles and metabolism (Lehti-Shiu and Shiu, 2012). PKs also participate in responses to drought, heat, and metal toxicity (Hasanuzzaman, 2020) and in the defense responses to herbivores and pathogens (Falco et al., 2001; Meng and Zhang, 2013). Several of these responses are predicted to become increasingly relevant in agriculture as a result of climate change; indeed, extreme temperatures and drought are obvious threats from global warming (Dai, 2013; Teixeira et al., 2013). Moreover, pest control is also prone to become more challenging with climate instability (Gregory et al., 2009). Therefore, the study of molecules and processes associated with both biotic and abiotic stresses is highly relevant to the current setting (Ahuja et al., 2010).

Dardick et al. (2007) noted that phylogenomic studies are particularly valuable in the analysis of large and conserved gene groups such as PKs because of their ability to form a basis for functional predictions and permit the identification of genes with unique properties, which can in turn allow rational selection of candidates for more detailed studies. The first works on the classification of PKs were based on the conservation and phylogenetic analyses of catalytic domains of eukaryotic proteins (Hanks and Hunter, 1995). Later studies also considered sequence similarity and domain structure outside the catalytic domains in categorization (Manning et al., 2002; Niedner et al., 2006). More recently, the availability of low-cost technologies for sequencing whole genomes have allowed the characterization of species’ kinomes, i.e., their entire repertoire of PKs. Compared to the human genome, plant genomes generally contain not only many more PK genes but also atypical kinase families – either exclusive to plant genomes or of prokaryotic origin (Zulawski and Schulze, 2015). This expansion likely resulted from segmental, whole-genome, and tandem duplication events (Hanada et al., 2008). *Arabidopsis thaliana* was the first plant to have its kinome compiled (Champion et al., 2004), followed by several other economically important species (Dardick et al., 2007; Liu et al., 2015; Zhu et al., 2018b).

Several studies have characterized kinases in sugarcane. The broadest study is likely that conducted by Papini-Terzi et al. (2005), who identified sequences corresponding to signal transduction components in the sugarcane expressed sequence tag database (Vettore et al., 2003). Although they obtained substantial results considering the limited resources available at the time, these researchers reported a relatively low number of PKs. Other studies have revealed that sugarcane PKs are involved in this plant’s development and response to environmental stimuli (Carraro et al., 2001; Pagariya et al., 2012; Li et al., 2017). Even more relevant is the compelling evidence that a leucine-rich repeat (LRR) receptor-like kinase is related to sucrose-accumulating sugarcane tissues and genotypes, which indicates its involvement in the regulation of sucrose synthesis (Vicentini et al., 2009). However, a more comprehensive characterization of sugarcane PKs has not yet been performed. Recently, a high-quality, chromosome-level genome assembly for sugarcane was made available (Zhang et al., 2018). The assembly of the genome of the *S. spontaneum* (Ssp) AP85-441 clone (2*n* = 4x = 32) is also allele-defined, i.e., it provides separate sequences of each of the four chromosome copies (haplotypes). Therefore, each gene can show up to four alleles (Zhang et al., 2018, 2019b; Li et al., 2020). The availability of this information-rich reference has since opened a range of possibilities in sugarcane research, such as the detailed characterization of specific groups of genes. Since polyploidy may result in chromosome rearrangements, gene loss, and unequal rates of sequence evolution and can favor gene neofunctionalization (Premachandran et al., 2011), the sugarcane genome provides fertile ground for related evolutionary and functional studies.

In this context, the main objective of this work was to identify and classify the complete set of PKs present in the Ssp and Sbi genomes. For this purpose, we performed phylogenetic analyses and *in silico* predictions of the properties of these proteins. Taking advantage of the completeness of the available information, we explored the impact of whole-genome and tandem duplications in the distribution and diversification of the genes encoding PKs in the genomes of the two plants. Finally, we constructed coexpression networks using RNA sequencing (RNA-Seq) to evaluate the expression of PK-encoding genes across different sugarcane and sorghum tissues and genotypes.

## Materials and Methods

### Kinase Identification and Domain Investigation

All kinase identification procedures were performed for both Sbi and Ssp. The Sbi protein-coding gene sequences and additional files from the Sbi genome (v3.1.1) were obtained from Phytozome v.13 (Goodstein et al., 2012). Ssp data were obtained from the AP85-441 genome (Zhang et al., 2018). The Ssp reference contains information at the allele level, considering the most conserved allele among all allelic copies and paralog/tandem duplications of each gene to represent it as a “gene model” (GM). In this assembly, the first part of a gene accession number refers to the GM; the last number refers to order of the allele; and the last letter may refer to the allelic copy (haplotypes A–D), it belongs to or indicate that the gene is tandemly (T) or dispersedly (P) duplicated. For Ssp, we identified PK genes in two ways: first, disregarding allelic relationships among genes; and secondly, considering their organization into GMs. All sequences obtained were aligned against the “typical” Pkinase (PF00069) and Pkinase_Tyr (PF07714) families with hidden Markov models (HMMs) retrieved from the Pfam database (El-Gebali et al., 2019) using HMMER v.3.3 (Eddy, 1998). An *E*-value cutoff of 0.1 was used, and we retained only sequences that covered at least 50% of the respective Pkinase domain. To avoid redundancy, we selected only the longest variant for genes with isoforms. The domain composition of the putative PKs was also investigated *via* the HMMER web server (Finn et al., 2011) and Pfam database. The distribution of PKs across the Sbi and Ssp chromosomes was visualized using MapChart v2.2 software (Voorrips, 2002).

### Subfamily Classification and Phylogenetic Analyses

All PKs identified were classified into subfamilies according to HMMs built based on a previous classification and analyses of kinases of 25 plant species (Lehti-Shiu and Shiu, 2012): *Aquilegia coerulea* (Aco), *Arabidopsis lyrata* (Aly), *A. thaliana* (Ath), *Brachypodium distachyon* (Bdi), *Carica papaya* (Cpa), *Citrus clementina* (Ccl), *Citrus sinensis* (Csi), *Chlamydomonas reinhardtii* (Cre), *Cucumis sativus* (Csa), *Eucalyptus grandis* (Egr), *Glycine max* (Gma), *Manihot esculenta* (Mes), *Medicago truncatula* (Mtr), *Mimulus guttatus* (Mgu), *Oryza sativa* (Osa), *Populus trichocarpa* (Ptr), *Prunus persica* (Ppe), *Physcomitrella patens* (Ppa), *Ricinus communis* (Rco), *Selaginella moellendorffii* (Smo), *Setaria italica* (Sit), *Vitis vinifera* (Vvi), *Volvox carteri* (Vca), *Zea mays* (Zma), and an earlier version of the Sbi genome, which we called v.1. Each Ssp and Sbi protein, previously identified as a putative kinase, was aligned against all these subfamily HMMs and considered as part of the top-scoring subfamily (*E*-value cutoff of 0.1). This classification was confirmed through phylogenetic analyses. The Pkinase domains of the putative PKs were aligned using Muscle v.3.8.31 (Edgar, 2004), and a phylogenetic tree was estimated using a maximum likelihood approach implemented in FastTreeMP v2.1.10 (Price et al., 2010) with 1,000 bootstrap replicates using the CIPRES gateway (Miller et al., 2010). Different trees were constructed for PKs from Sbi; PKs from Ssp; and PKs from both Sbi and Ssp. The dendrogram was visualized using R statistical software (R Core Team, 2013) with the ggtree (Yu et al., 2017) and ggplot2 (Villanueva and Chen, 2019) packages.

### Kinase Characterization

For each PK identified, the following characteristics were determined: gene chromosomal location and intron number, using GFF files; predicted subcellular localization, with WoLF PSORT (Horton et al., 2007), CELLO v.2.5 (Yu et al., 2006) and LOCALIZER v.1.0.4 (Sperschneider et al., 2017) programs; presence of N-terminal signal peptides, using SignalP v.4.1 Server (Petersen et al., 2011); presence of transmembrane domains, using TMHMM v.2.0 Server (Krogh et al., 2001); and Gene Ontology (GO) categories (Ashburner et al., 2000), using the Blast2GO tool (Conesa et al., 2005) with the SWISS-PROT (Bairoch and Apweiler, 2000) and UniProt (UniProt Consortium, 2007) databases. For Sbi PKs, alternative splicing (AS) events were investigated using the Plant Alternative Splicing Database (Min, 2013; Min et al., 2015). The comparison of these characteristics and calculation of descriptive statistics were performed with R. Analysis and visualization of GO categories were performed using the REViGO tool (Supek et al., 2011).

### Duplication Events

To investigate PK duplication events, we used the Multiple Collinearity Scan (MCScanX) toolkit (Wang et al., 2012). Tandem duplications were visualized with MapChart, and segmental events were visualized with Circos software (Krzywinski et al., 2009). Synonymous substitution (*Ks*) and nonsynonymous substitution (*Ka*) rates were also estimated for segmental duplications using MCScanX (Wang et al., 2012), and *Ks* values were used to estimate the date of duplication events: T = *Ks*/2*λ*, where *λ* is the mean value of the clock-like rates of synonymous substitutions (6.5 × 10^−9^; Gaut et al., 1996).

### RNA-Seq Experiments

Data from several RNA-Seq experiments were used to estimate kinase expression, as summarized in Table 1. Sbi datasets were retrieved from NCBI’s Sequence Read Archive (SRA; Leinonen et al., 2010) and are described in Supplementary Table 1. Samples from different tissues and varieties were used (Dugas et al., 2011; Freeling et al., 2015; Makita et al., 2015; Kebrom et al., 2017; Varoquaux et al., 2019). To analyze sugarcane kinase expression, we used one published and three novel RNA-Seq datasets described in the following section.

**Table 1:** Information on samples used for RNA sequencing (RNA-Seq) experiments.

Sugarcane		
Species	Genotype	Tissue
*S. officinarum*	Badila De Java	Culms
*S. officinarum*	Criolla Rayada	Leaves
*S. officinarum*	White Transparent	Leaves
*S. robustum*	IJ76-318	Leaves
*S. spontaneum*	IN84-58	Culms, leaves
*S. spontaneum*	IN84-88	Leaves
*S. spontaneum*	Krakatau	Culms, leaves
*S. spontaneum*	SES205A	Leaves
*Saccharum* sp.	F36-819	Culms
*Saccharum* sp.	IACSP93-3046	Culms
*Saccharum* sp.	R570	Culms
*Saccharum* sp.	RB72454	Leaves
*Saccharum* sp.	RB855113	Roots
*Saccharum* sp.	RB855156	Leaves
*Saccharum* sp.	RB855536	Roots
*Saccharum* sp.	RB867515	Roots
*Saccharum* sp.	RB92579	Roots
*Saccharum* sp.	SP79-1011	Roots
*Saccharum* sp.	SP80-3280	Culms, leaves, roots
*Saccharum* sp.	TUC71-7	Leaves
*Saccharum* sp.	US85-1008	Leaves
**Sorgum**		
**Species**	**Genotype**	**Tissue**
*Sorghum bicolor*	BTX623	Pollen, microspore, roots, ligule epidermial tissue, blade epidermial tissue
*Sorghum bicolor*	BTX623	Roots, shoots
*Sorghum bicolor*	BTX623	Seeds, spikelets, top internodes
*Sorghum bicolor*	BTX642	Leaves, roots
*Sorghum bicolor*	R.07020	Internodes 1–4
*Sorghum bicolor*	RTX430	Leaves, roots

#### Sugarcane Plant Material and RNA-Seq

Sugarcane hybrids and *S. officinarum* and Ssp clones were used for expression analyses in sugarcane. Four independent experiments were performed and are detailed in Supplementary Table 2. Experiment 1 was based on root material from the RB867515, RB92579, RB855113, RB855536, SP79-1011, and SP80-3280 hybrid cultivars. This trial was carried out in a greenhouse with three replicates per cultivar in a completely randomized design. Plants were grown in 18-L plastic pots with a mixture of 20% commercial planting mix and 80% sand. Ninety-five days after planting, we sampled the root material of each plant, avoiding tiller roots.

Experiments 2 and 3 were performed with leaf and culm (internode 1) samples, respectively, from plants grown in the field in Araras, Brazil (22° 18' 41.0'' and 23' 05.0'' W, at an altitude of 611 m). Leaf samples were collected from portions of the top visible dewlap leaves (+1) of 6-month-old sugarcane plants in April 2016. We collected the middle section of each leaf, removing the midrib. For culms, samples from the first internode were collected at four time points in 2016: April (synchronous with leaf sampling), June, August, and October.

In Experiment 2, described by Correr et al. (2020), we used samples from the SP80-3280, RB72454, and RB855156 hybrid cultivars; TUC71-7 and US85-1008 hybrids; White Transparent and Criolla Rayada *S. officinarum* genotypes; IN84-58, IN84-88, Krakatau, and SES205A Ssp genotypes; and IJ76-318 *Saccharum robustum* genotypes. For six genotypes – SP80-3280, RB72454, US85-1008, White Transparent, IN84-58, and SES205A – we collected and sequenced three biological replicates, while the others were represented by one biological replicate; all samples were represented by three technical replicates. Leaf samples were sequenced in two lanes. In Experiment 3, culm samples were collected from the SP80-3280 and R570 hybrid cultivars, F36-819 hybrid, and IN84-58 *S. spontaneum* genotype. Culm samples were sequenced in six lanes.

Experiment 4 was based on samples from the SP80-3280 and IACSP93-3046 hybrid cultivars, Badila De Java *S. officinarum* genotype, and Krakatau Ssp genotype. RNA samples were extracted in triplicate from the top (internode 3) and bottom (internode 8) culms and collected in the field in Ribeirão Preto, Brazil (21° 12' 28.7'' S, 47° 52' 29.1'' W) in June 2016.

After collection, samples were immediately frozen in liquid nitrogen and stored at −80°C until processed. Total RNA was extracted from 200 mg of ground roots and 50 mg of ground leaves or culms using an RNeasy Plant Mini Kit (Qiagen, Valencia, CA, United States). We quantified the RNA and verified its integrity in a 2100 BioAnalyzer using a Eukaryote Total RNA Nano Assay (Agilent Technologies, Santa Clara, CA, United States). A total of 300 ng of RNA per sample was used to prepare cDNA libraries with a TruSeq Stranded mRNA LT Kit (Illumina, San Diego, CA, United States). Libraries were sequenced on a HiSeq 2500 platform (Illumina, San Diego, CA, United States).

### RNA-Seq Data Processing and Coexpression Network Construction

The quality of the RNA-Seq data was assessed using FastQC software (Andrews, 2010). For read filtering and adapter removal, we used Trimmomatic v.0.39 (Bolger et al., 2014). In the Sbi and sugarcane datasets, bases with Phred scores below 20 were removed, and reads shorter than 30 bp were filtered out. In the sugarcane datasets, we also removed the first 12 bases of each read and increased the filter length to 75 bp. For transcript quantification, we used the DNA coding sequences (CDSs) from each species as reference, with k-mers of lengths 31 and 17 for Ssp and Sbi, respectively, in Salmon v.1.1.0 software (Patro et al., 2015). PK expression quantification was evaluated with transcripts per million (TPM) values. Heatmaps visualizing the expression of kinase subfamilies among tissues and cultivars were generated using the R package pheatmap (Kolde and Kolde, 2015) with average TPM values and a complete-linkage hierarchical clustering approach based on Euclidean distances.

Coexpression networks were estimated for PK subfamilies using a minimum Pearson correlation coefficient of 0.6 between PK quantifications across different subfamilies. Network analyses were performed using the R package igraph (Csardi and Nepusz, 2006). To assess the Ssp and Sbi network structures, hub scores for each subfamily were calculated considering Kleinberg’s hub centrality scores (Kleinberg, 1999), edge betweenness values estimated by the number of geodesics passing through the edge (Brandes, 2001), and communities defined using a propagating label approach (Raghavan et al., 2007).

## Results

### Genome-Wide Identification of PKs in Sugarcane and Sorghum

**Figure 1:**
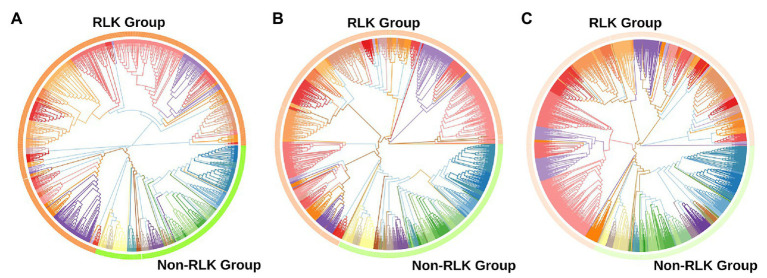
Phylogenetic analyses of putative protein kinases (PKs) identified in the *Saccharum spontaneum* (Ssp) and *Sorghum bicolor* (Sbi) genomes. **(A)** Phylogenetic tree of the 1,210 Sbi PKs organized in 120 subfamilies represented by different colors. **(B)** Phylogenetic tree of the 2,919 Ssp PKs organized in 119 subfamilies. **(C)** Phylogenetic tree of PKs in both Sbi and Ssp.

Using the established bioinformatic pipeline, we identified 2,919 putative Ssp and 1,210 putative Sbi PKs (Supplementary Tables 3 and 4), which were classified into groups and subfamilies according to the top-scoring correspondence to HMMs of 25 plant species (Lehti-Shiu and Shiu, 2012; Supplementary Tables 5 and 6) and phylogenetic approaches (Supplementary Figures 1–3), as fully described in Supplementary Results (Genome-Wide Identification of PKs in Sugarcane and Sorghum section). Similar to other kinomes (Singh et al., 2014; Wei et al., 2014; Zulawski et al., 2014; Liu et al., 2015; Yan et al., 2017, 2018; Zhu et al., 2018a,b), the most abundant group in both species was the receptor-like kinase (RLK)-Pelle group (Figure 1), which accounted for ∼70% of the PKs, followed by the calcium- and calmodulin-regulated kinase (CAMK); cyclin-dependent kinase, mitogen-activated protein kinase, glycogen synthase kinase, and cyclin-dependent kinase-like kinase (CMGC); tyrosine kinase-like kinase (TKL); serine/threonine kinase (STE); and cyclic AMP-dependent protein kinase (cAPK), cGMP-dependent protein kinase, and lipid signaling kinase families (AGC); and casein kinase 1 (CK1) groups. This expansion in the Sbi and Ssp kinomes is apparently related to a few specific families within the group, most notably LRR, RLCK, DLSV, L-LEC, and SD-2b. These families have been associated with the increased number of kinases in RLK-Pelle (Zhu et al., 2018a,b), mostly because of their relationship to biotic and abiotic stress responses (Dezhsetan, 2017). The subfamily abundances obtained for Ssp and Sbi were similar, and only the pancreatic eukaryotic initiation factor-2alpha kinase (PEK_PEK) subfamily was exclusive to Sbi.

We compared the subfamily quantities to other plant species (Lehti-Shiu and Shiu, 2012). The heatmap (Supplementary Figure 4) visualizing the similarities in the numbers of PKs indicated a closeness between the Ssp and Sbi kinase compositions; however, both exhibited closer relationships with other species than with each other. The dendrogram constructed based on the columns (plant species) enabled the identification of the species most similar to Sbi and Ssp in terms of PK quantities. Sbi was found to belong to a cohesive clade with Zma, Bdi, and Sbi v.1, and Ssp belonged to a clade with Sit and Osa. Interestingly, although the two groups were separated by other species, together, they corresponded to all of the monocotyledon species under comparison.

**Figure 2:**
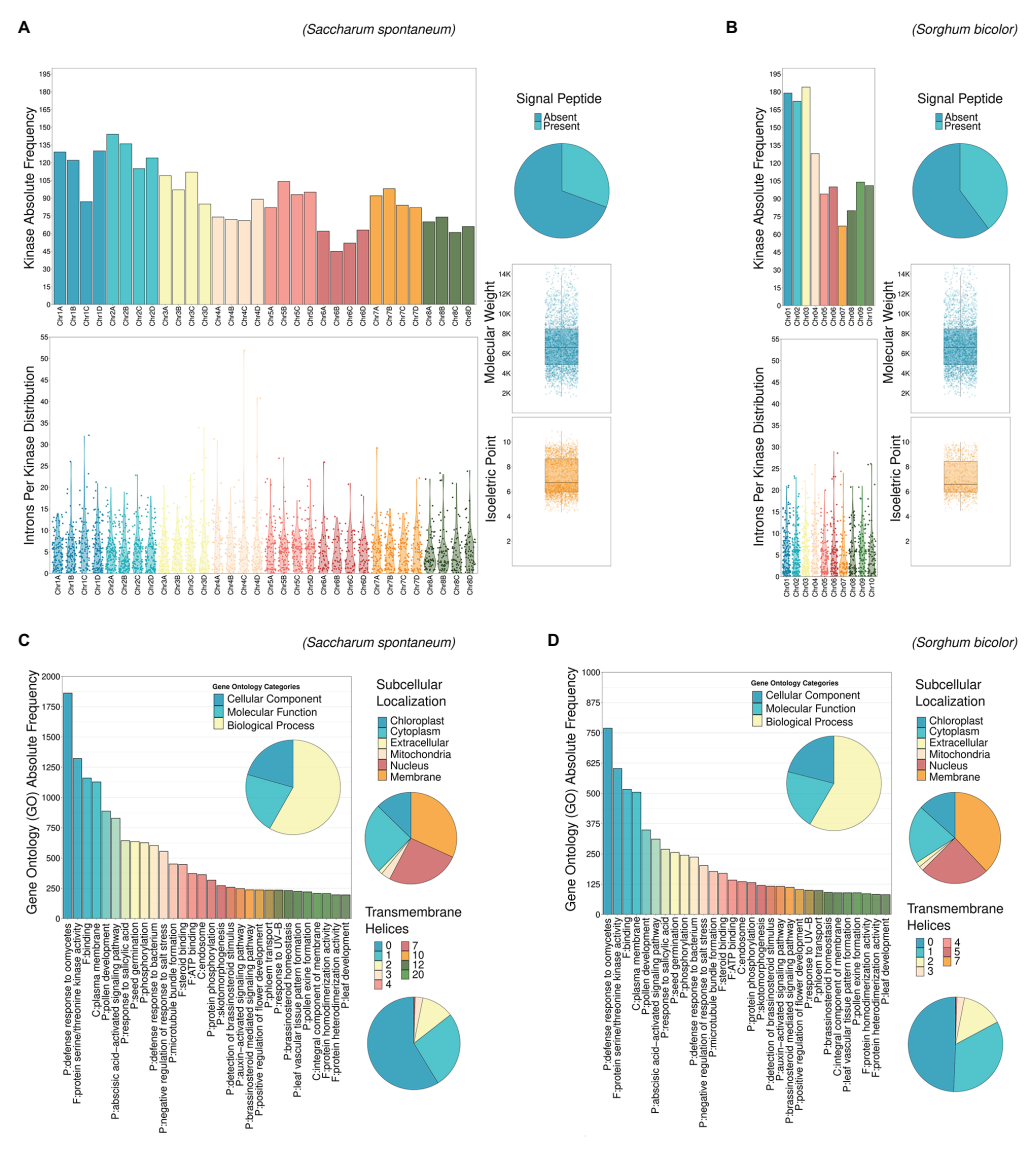
Descriptive analysis of kinase characteristics in Ssp **(A,C)** and Sbi **(B,D)**: chromosomal distribution, intron length and chromosomal occurrence, presence of signal peptides, molecular weights (MWs), isoelectric points (pIs), Gene Ontology (GO) terms, subcellular localization, and presence of transmembrane helices.

A full characterization of Ssp and Sbi PKs is described in Supplementary Results (Characterization of PKs section) and summarized in Figure 2, including their chromosome distribution (Supplementary Tables 8 and 9), intron organization (Supplementary Tables 10 and 11), domain composition (Supplementary Tables 12–17), GO annotation (Supplementary Tables 18 and 19; Supplementary Figure 5) and protein properties, including the presence of signal peptides and transmembrane helices in the PKs, their estimated molecular weights (MWs), theoretical isoelectric points (pIs), and subcellular localizations (Supplementary Tables 20 and 21). All these attributes of PKs are summarized at subfamily level in Supplementary Tables 22 (Sbi) and 23 (Ssp), with domain composition described in Supplementary Tables 24 and 25.

### Kinase Duplication Events in Sugarcane and Sorghum

**Figure 3:**
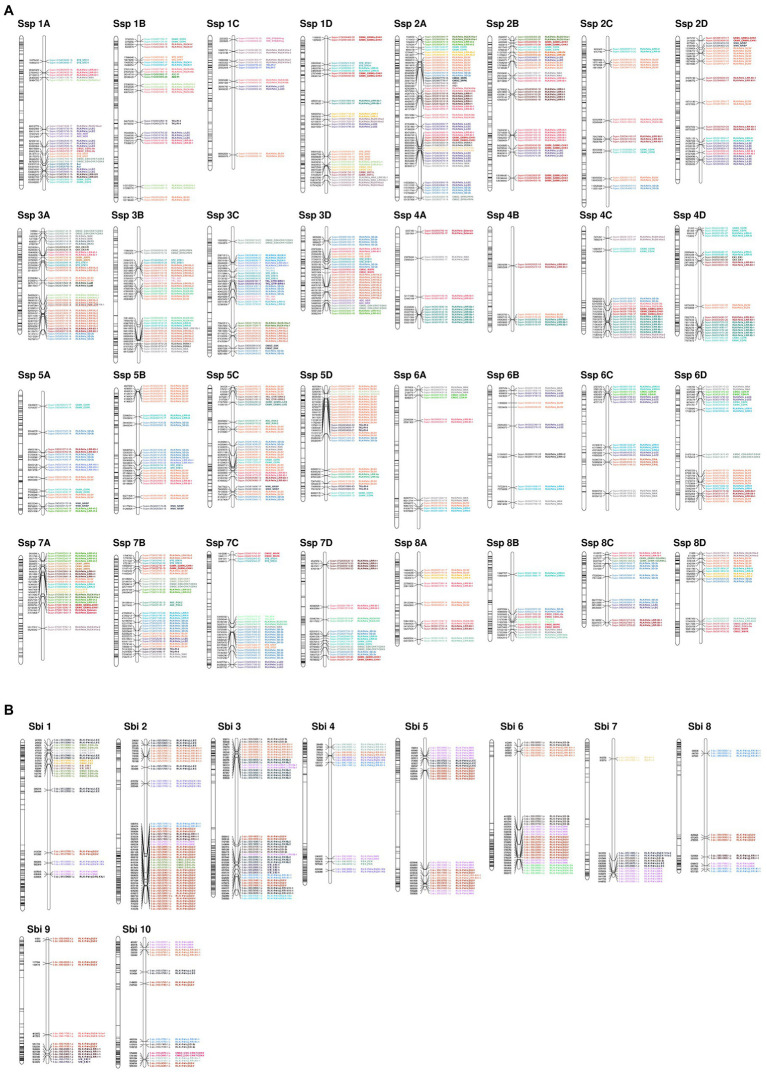
Kinase distribution along **(A)** *S. spontaneum* and **(B)** *S. bicolor* chromosomes. For each chromosome, all genes with kinase domains are indicated on the left, and only the tandemly organized kinases are indicated on the right, colored and labeled according to the subfamily classification.

**Figure 4:**
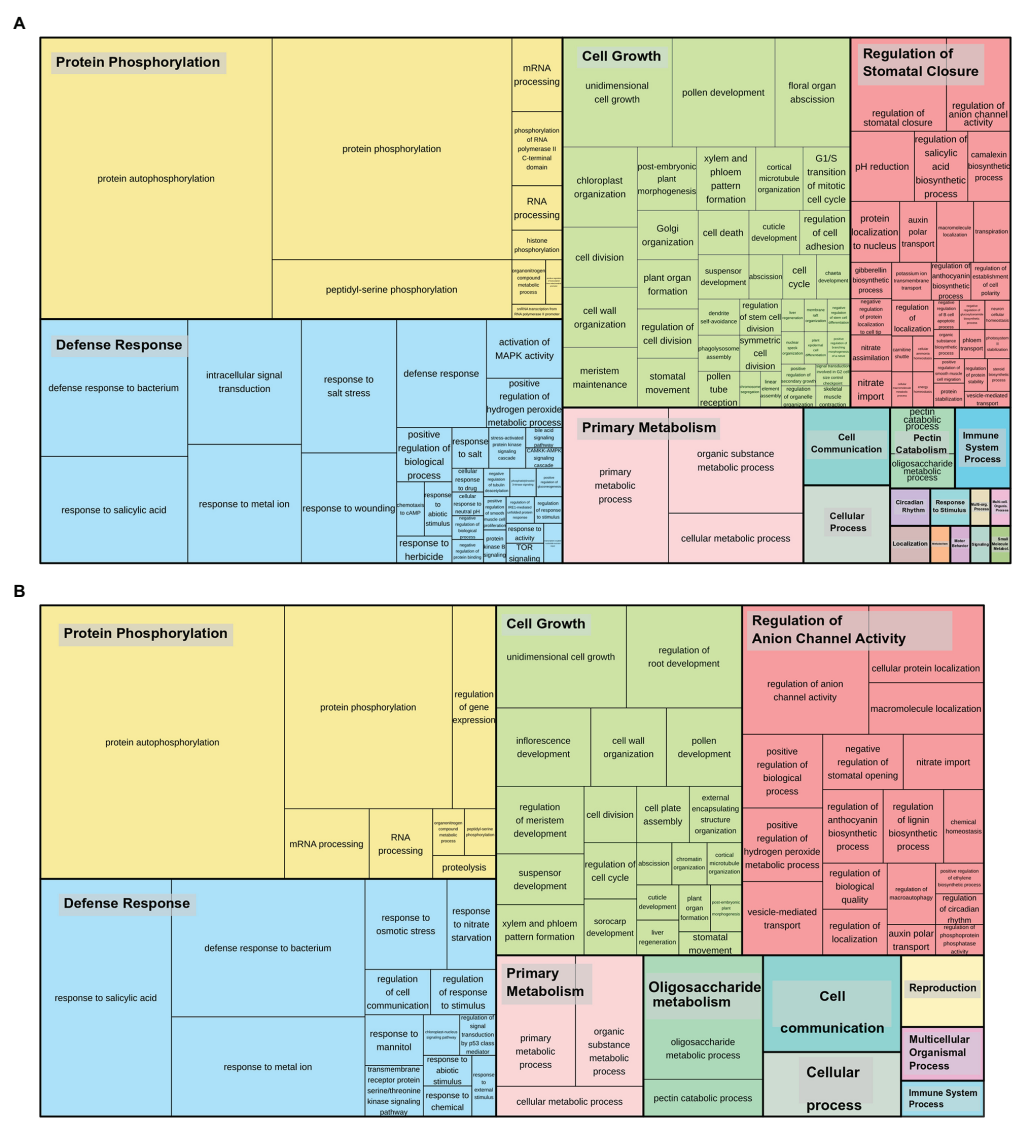
Gene Ontology (GO) categories (biological processes) related to tandemly duplicated kinases in **(A)** *S. spontaneum* and **(B)** Sbi. The size of the subdivisions within the blocks represents the abundance of that category in this set of kinases. The colors are related to the similarity to a representative GO annotation for the group.

Gene duplications in Sbi and Ssp kinases were investigated using MCScanX. We identified numerous kinase genes (1,165 in Sbi and 2,919 in Ssp) that originated from dispersed (7.73% in Sbi and 1.68% in Ssp), proximal (3.18% in Sbi and 1.88% in Ssp), tandem (10.04% in Sbi and 8.94% in Ssp), and segmental duplications (78.97% in Sbi and 87.43% in Ssp; Supplementary Tables 26 and 27). Ssp PKs with origins related to tandem duplications were unevenly distributed across all allele copies on chromosomes (ranging from two events on Chr4-B to 16 events on Chr3-A). All Sbi chromosomes contained PKs that originated from tandem duplications, ranging from one PK in chromosome 4 to 26 on both chromosomes 2 and 3 (Figure 3A). By analyzing the tandemly duplicated PKs within subfamilies, we found 19 subfamilies containing PKs that originated from tandem duplication. The highest percentages of such Sbi PKs were found in the RLK-Pelle_RLCK-Os, RLK-Pelle_LRR-I-1, CMGC_CDKL-Os, RLK-Pelle_LRK10L-2, and CMGC_CK2 subfamilies (Figure 3B). In Ssp 64 subfamilies had tandemly duplicated PKs, and the five subfamilies with the highest percentages were RLK-Pelle_RKF3, RLK-Pelle_LRR-VIII-1, CAMK_CAMK1-DCAMKL, RLK-Pelle_LRR-XIIIb, and TKL_Gdt. In Ssp the distribution of PKs did not exhibit a clear pattern along chromosomes (Figure 3A). In Sbi, these were concentrated in subtelomeric regions and were almost nonexistent in pericentromeric regions (Figure 3B). This pattern was observed more clearly when only tandemly distributed Sbi PKs were considered. We also performed a GO analysis to determine the categories related to tandemly duplicated kinases. The GO terms describing the biological processes of these proteins were similar between Ssp and Sbi (Figures 4A,B), and considerable correspondences with the total number of GO terms related to the entire set of PKs were observed (Supplementary Figures 5A,B).

Segmental duplications accounted for the highest percentage of identified duplication types in both Sbi and Ssp PKs. The highest quantities in Ssp were observed in the allelic copies of chromosomes 1 and 2, which also contained the most PKs. In Sbi, chromosome 1 exhibited the most segmental duplications, although chromosome 3 had the most PK genes. For all gene pairs within these collinear duplications, we calculated the *Ka* and *Ks* values to obtain a time indicator of these events and evaluated the primary influence of PK expansion by calculating the *Ka*/*Ks* ratio. We considered each gene pair to be under neutral (*Ka*/*Ks* = 1), negative (*Ka*/*Ks* < 1) or positive selection (*Ka*/*Ks* > 1; Zhang et al., 2006). *Ks* values were clearly more evenly distributed in Sbi than in Ssp (Supplementary Figures 6A,B and Supplementary Tables 28 and 29), which had 1,287 (27.5%) segmentally duplicated PKs with a *Ks* of < 0.05. We used *Ks* values to estimate the occurrence times of these duplications; the times ranged between 0 and 230.1 million years ago (MYA) in Ssp with an average of 45.6 MYA, and between 4.9 and 229.7 MYA in Sbi, with an average of 96.8 MYA. Most segmental duplications with *Ks* < 0.05 in Ssp were estimated to have occurred less than 3.83 MYA. Regarding the *Ka*/*Ks* ratio, we found the largest percentage of gene pairs as likely to be under negative selection in both species (∼86% in Ssp and ∼88% in Sbi).

**Figure 5:**
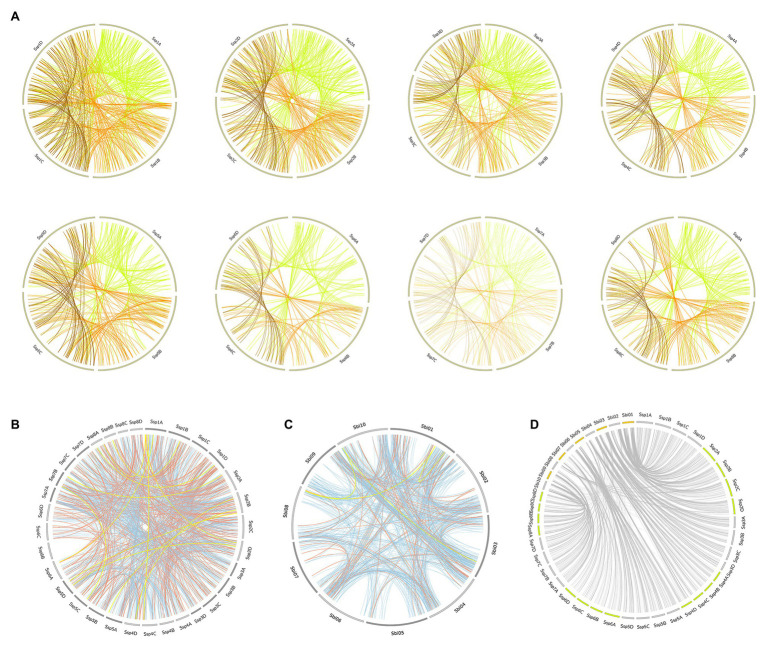
Segmental duplication events in the Ssp and Sbi genomes, divided into **(A)** Ssp duplications between alleles on the same chromosome, with the colors representing the origin of the duplication (green for allele **A**, orange for allele **B**, and brown for allele **C**). **(B)** Ssp duplications between chromosomes, excluding events between alleles on the same chromosome; and **(C)** Sbi duplications. The colors in **(B,C)** indicate the selection type of the gene pair duplication (orange indicates negative selection; light blue, positive selection; and yellow, neutral selection). **(D)** Representation of kinase correspondences between Sbi and Ssp., indicating the synteny relationships among these species.

The segmental events among alleles had different configurations, but in most duplications, the order of PKs on one allele was retained on the other allele (Figure 5A). Syntenic blocks between chromosomes were much more frequent in Ssp (Figure 5B) than in Sbi (Figure 5C), mainly due to the allele specificity of Ssp. The duplication patterns were similar between Ssp and Sbi, as shown in Figure 5D, where the kinase genomic correspondences indicate the increased synteny between the two species. In most subfamilies, the origin of most PKs was characterized by segmental duplications (109 and 115 subfamilies in Sbi and Ssp respectively; Supplementary Tables 22 and 23). Interestingly, four subfamilies in Ssp and eight subfamilies in Sbi did not contain any PKs that originated from segmental duplications (Supplementary Tables 22 and 23).

**Figure 6:**
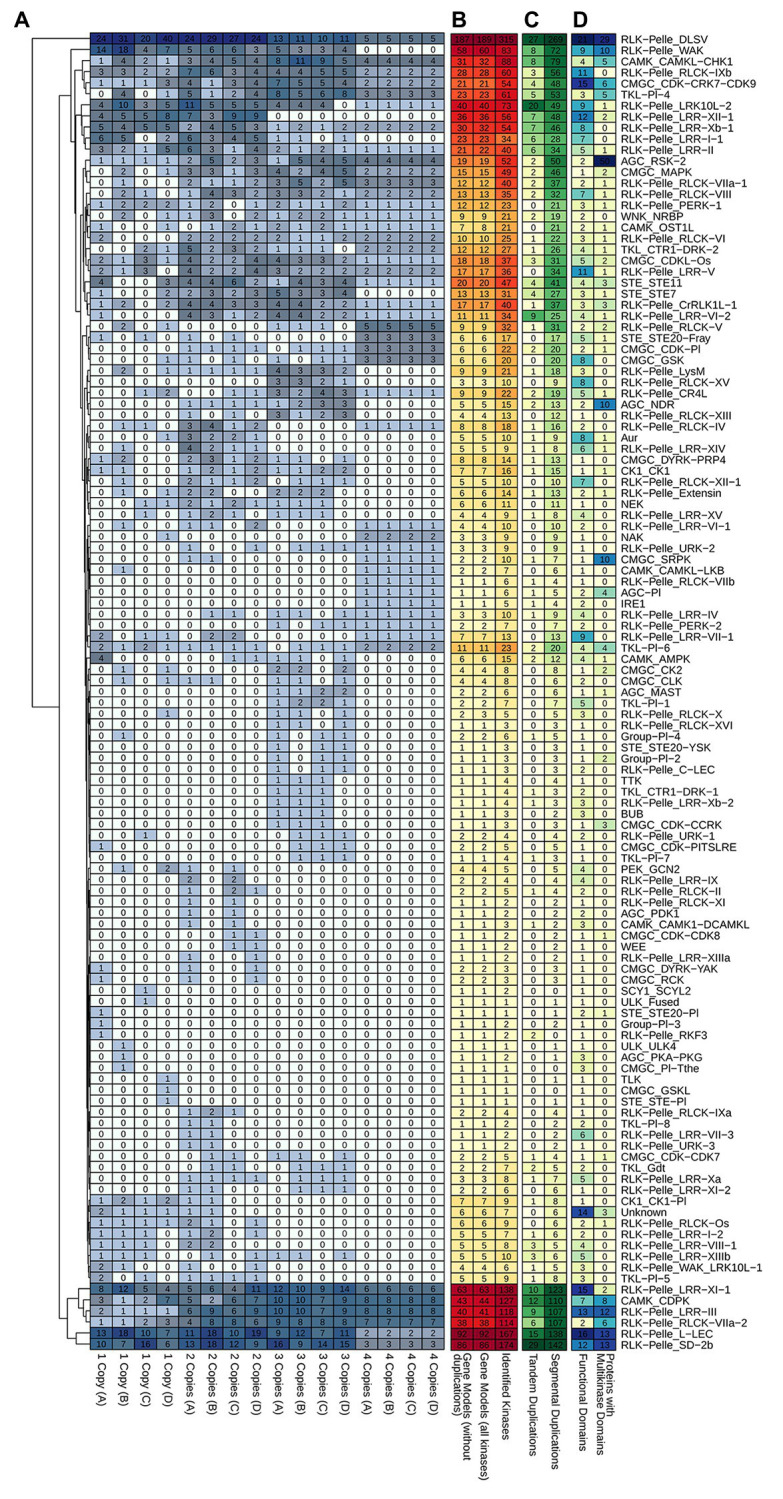
Heatmap representations of kinase subfamily profiles in *S. spontaneum* related to **(A)** kinase copies among alleles. **(B)** Subfamily quantification considering the entire set of kinases and the respective quantity of gene models. **(C)** Tandem and segmental duplication events; and **(D)** the presence of different functional domains and multikinase domain-containing proteins within subfamilies.

For Ssp we performed additional analyses to assess the distribution of kinase copies among alleles and investigated possible associations among alleles, duplications, and domains (Figure 6). Each Ssp GM can have up to four allelic copies, depending on the genomic organization of the gene. Subfamilies with larger numbers of PKs had a more dispersed organizational profile in terms of the number of allelic copies per GM. Subfamilies with fewer GMs, on the other hand, did not have a uniform configuration. These subfamilies constitute the majority of the Ssp kinome [∼60% of the subfamilies had five or fewer representative GMs, and 33 subfamilies (∼30%) had only one GM]. Even with the few related proteins, these small subfamilies did not exhibit similar characteristics. Only three of these GMs had copies on the four alleles, whereas 10, 9, and 11 GMs contained copies on three alleles, two alleles, and only one allele (three in allelic model A, three in B, two in C, and three in D), respectively. More tandem and segmental duplications were observed in subfamilies with more elements, but this pattern did not hold for the quantity of functional domains and multikinase domains (Figure 6).

### Estimates of Kinase Expression and Construction of Coexpression Networks

Quantification of kinase expression in Sbi and Ssp was performed *via* a wide variety of datasets comprising different tissues and genotypes. From the CDS quantifications, we separated the subset of kinase coding genes. *Via* TPM values, Sbi kinase expression was quantified in 205 samples (Supplementary Table 30); Ssp kinase expression, in 234 (Supplementary Table 31). To quantify expression at the subfamily level, the TPM values for all PK members in a subfamily were averaged in each sample (Supplementary Tables 32 and 33). However, most experiments contained several replicates, and the sample TPM values were also averaged to separately represent the unique characteristics of a tissue from a specific genotype (Supplementary Tables 34 and 35).

**Figure 7:**
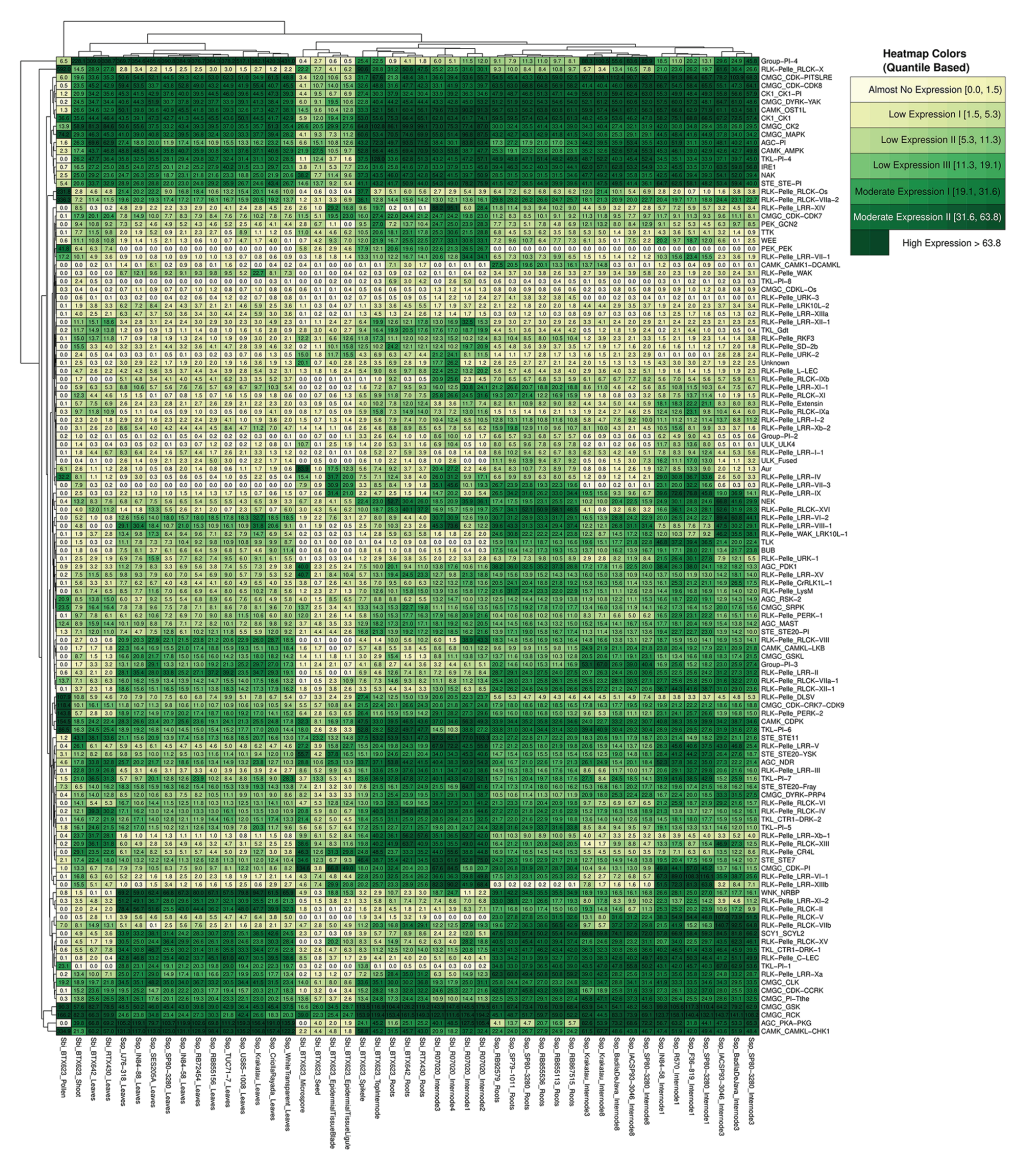
RNA expression profiles of *S. spontaneum* and *S. bicolor*, shown on a heatmap indicating the average sample values of different combinations of genotypes and tissues (columns) and considering the organization of kinase subfamilies (rows).

The expression quantification of Ssp and Sbi kinase subfamilies was visualized with a heatmap (Figure 7). There was a noticeable division of the columns into five groups, as was also identified by the total within sum of squares using a range of group configurations (2–10). The groups were separated into sugarcane samples from internodes and roots; Sbi samples from internodes, roots, and spikelets; Sbi samples from epidermal tissues, seeds, and microspores; Sbi and sugarcane samples from leaves and shoots; and Sbi samples from pollen. The expression patterns of kinase subfamilies were more similar among similar tissues from different species. However, these clusters contained subdivisions supporting the species specificities.

The differences in subfamily expression profiles were investigated further. For each subfamily, we calculated the dispersion of expression among genotypes and tissues using the SD and coefficient of variation (Supplementary Tables 36 and 37). The divergence of these measures among tissues was high in Sbi, as observed in the heatmap and indicated by the high values of the coefficient of variation (ranging from ∼38 to ∼297%). In Ssp on the other hand, 16 subfamilies exhibited relatively uniform expression patterns in the analyzed samples (with coefficient of variation of less than or equal to 20%). This difference is possibly explained by the greater diversity of tissues used for Sbi than for Ssp. To identify subfamilies with the highest and lowest expression values, we calculated additional statistical measures to summarize the distribution of TPM values in each subfamily (i.e., minimum, maximum, mean, and 1st, 2nd, and 3rd quartiles). We selected 12 subfamilies (10% of the dataset) with the highest and lowest values of all these measures. We considered a subfamily as having the highest or lowest expression values if that subfamily was ranked in at least four of the six measures. We identified 8 and 12 subfamilies in Sbi and Ssp respectively, with the highest expression patterns in the dataset. Surprisingly, three of these subfamilies (CK1_CK1, CMGC_GSK, and CMGC_RCK) had the highest expression value in both species. Importantly, in addition to being overexpressed in Sbi, the members of the CMGC_GSK subfamily also contain many functional domains, which might reflect their high expression. Another overexpressed subfamily in the Sbi kinome (but not in Ssp) was CMGC_MAPK, which has previously been associated with stress signaling in Ssp and Sbi (Zhang et al., 2015; Li et al., 2016b; Paungfoo-Lonhienne et al., 2016; Srivastava and Kumar, 2020; Tuleski et al., 2020; Wang et al., 2020). Although their expression values were significantly increased, these subfamilies did not contain the highest numbers of kinases.

Using this approach, eight and nine subfamilies in Ssp and Sbi, respectively, with the lowest expression values were identified with two overlaps (CMGC_CDKL-Os and RLK-Pelle_URK-3). RLK-Pelle_URK-3 had only one kinase member in both the Sbi and Ssp kinomes; however, CMGC_CDKL-Os had 37 and 23 kinases in Ssp and Sbi, respectively. The apparent lack of a correlation between the expression values and the numbers of kinases in the subfamilies was also evidenced by any combination of genotype/tissue with a significant Spearman correlation coefficient (Supplementary Tables 38 and 39).

**Figure 8:**
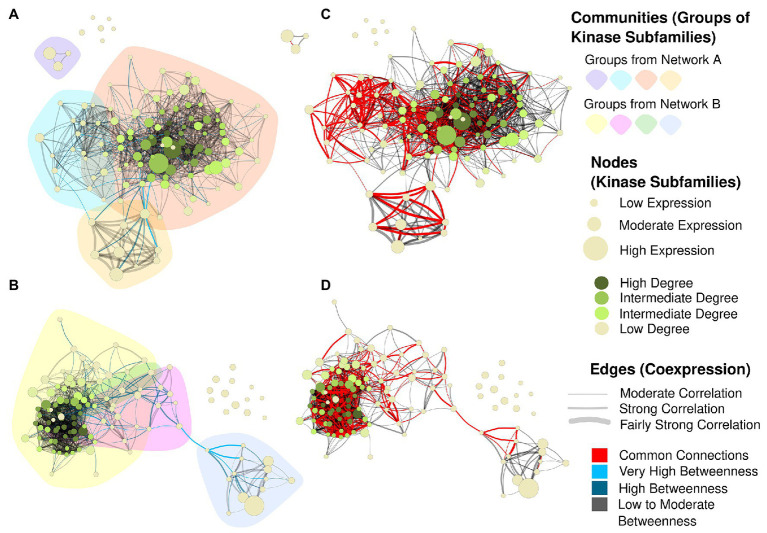
Coexpression networks for Sbi and Ssp kinase subfamilies. Each node corresponds to a different subfamily, its size corresponds to the average expression value for all kinases within the subfamily in different samples, and its color corresponds to the hub score and ranges from beige to dark green. Each edge corresponds to a correlation with a Pearson correlation coefficient of at least 0.6. The correlation strength is represented by the edge’s width and the edge betweenness score is represented by the color (ranging from black to light blue, with light blue representing the highest values). **(A)** Sbi network with the background colored according to the community detection analysis. **(B)** Sbi network indicating the similarities with the Ssp network in red. **(C)** Ssp network with community structure information. **(D)** Ssp network indicating the similarities with the Sbi network in red.

Together, the dendrogram and the heatmap indicate the presence of groups of subfamilies with high similarities, whose expression patterns changed jointly according to the tissue/genotype. Collectively considering all Sbi and Ssp quantifications, we evaluated their similarities through correlation analysis. The strongest correlations were higher than 0.97 for the two subfamily pairs RLK-Pelle_RLCK-Os/RLK-Pelle_RLCK-VIIa-2 and RLK-Pelle_RLCK-VIIa-2/RLK-Pelle_RLCK-X. To complement the assessment of the similarities in RNA expression among the subfamilies, we constructed coexpression networks for each species based on the expression correlation among samples in each subfamily (Figure 8). Each node in the network represents a different subfamily (the node sizes represent the mean of the expression values within the subfamily), and each connection has a minimum Pearson correlation coefficient of 0.6 (the edge sizes represent the degree of the correlation). Based on the network structure, we evaluated the presence of cohesive clusters formed by correlated subfamilies using a network community detection approach. In the Sbi network (Figure 8A), we identified four different modules with 87, 15, 3, and 9 elements. Four modules were also identified in the Ssp network (Figure 8C), but the distribution of the elements differed (83, 13, 8, and 2). In both networks, the remaining subfamilies (six in the Sbi network and 13 in the Ssp network) were identified as disconnected elements, and there was no evident similarity between communities (Supplementary Figure 7; Supplementary Tables 40 and 41).

The Sbi and Ssp networks exhibited many different forms and structures; however, by highlighting the connections in common between such networks (Figures 8B,D), we observed a similar substructure between representations. The main network components were connected by this core structure, indicating the strongest correlations between kinase subfamilies. The Ssp network (Figure 8D) contained an edge that clearly separates the network into two components; interestingly, this edge also belonged to the common structure. By coloring the network edges according to the betweenness measure (Figures 8A,B), we defined the connections between subfamilies that were most likely to represent vulnerabilities in the networks, possibly indicating influential subfamilies in this complex system. The most important connections were related to the subfamily pairs CAMK_CDPK/RLK-Pelle_LRR-VI-2 and CAMK_CDPK/CMGC_RCK in Sbi, and to RLK-Pelle_L-LEC/RLK-Pelle_LRR-VIII-1, RLK-Pelle_CR4L/RLK-Pelle_LRR-VIII-1, and RLK-Pelle_CR4L/RLK-Pelle_LRR-Xb-1 in Ssp (Supplementary Tables 42 and 43).

The most influential subfamilies in the networks were defined by ranking the nodes according to their hub scores, which were used to color the nodes in the network, and highest hub scores denote kinase subfamilies with the most connections (Supplementary Figures 8, 9; Supplementary Tables 40 and 41). The top five scores belonged to the subfamilies RLK-Pelle_LRR-III, RLK-Pelle_RLCK-XII-1, CMGC_CDK-CRK7-CDK9, CMGC_GSK, and RLK-Pelle_Extensin in Ssp., and to the Unknown category, RLK-Pelle_LRR-XV, CMGC_GSK, STE_STE20-Fray, and CAMK_OST1L in Sbi. Additionally, high expression values in subfamilies did not indicate increased hub scores (Figure 8).

## Discussion

Sugarcane possesses one of the most complex genomes known among crops (De Souza Barbosa et al., 2020). Only in 2018, modern technologies enabled the assembly of a chromosome- and allele-level genome of an Ssp clone (Zhang et al., 2018). This study paved the way for several comprehensive analyses of gene families in the species (Hu et al., 2018; Shi et al., 2019; Wang et al., 2019a,b,c; Zhang et al., 2019b; Huang et al., 2020; Li et al., 2020; Su et al., 2020a). Here, we analyzed the kinome of not only sugarcane but also sorghum, a close diploid relative. Studies estimate that the *Saccharum* and *Sorghum* lineages diverged 4.6–5.4 MYA (Kim et al., 2014). After diverging from *Miscanthus* 3.1–4.6 MYA (Kim et al., 2014), the *Saccharum* lineage experienced at least two rounds of whole-genome duplication (WGD; Zhang et al., 2018), whereas Sbi remained diploid. Therefore, sorghum genomic resources have been extensively employed in sugarcane studies (Okura et al., 2016; Mancini et al., 2018; Bedre et al., 2019). As the genomes of both species are now available, comparisons of the diversity, organization and expression of PKs between the two species enable us to perform in-depth explorations of the evolutionary history of these proteins.

### Expansion and Diversification of Sugarcane and Sorghum Kinomes

Lehti-Shiu and Shiu (2012) indicated that substantial numerical variations in the PK superfamily exist among plant species; however, this variation could be overestimated due to differences in the completeness of the genomic assemblies. Moreover, the estimates presented by this and other studies (Singh et al., 2014; Wei et al., 2014; Liu et al., 2015; Zhu et al., 2018a,b) indicated a number of PKs in Sbi (1,210) very similar to those of other Poaceae species, which range between 1,041 in Bdi and 1,417 in Osa (Lehti-Shiu and Shiu, 2012; Wei et al., 2014). The Ssp genome, on the other hand, contains one of the largest numbers of PK genes reported for any plant species (2,919), ranking below only the allohexaploid genome of *Triticum aestivum* (3,269 PKs; Yan et al., 2017). However, we must consider that this identification was performed using a genome with allele-level information; when only Ssp GMs (i.e., single representatives of all copies of a gene) were analyzed, we found a much lower number of PK genes (1,345), which is also within the range of PKs in other Poaceae species. This discrepancy reinforces the hypothesis of Lehti-Shiu and Shiu (2012) that the expansion of PK genes is directly related to recent WGD events, a suggestion that was made considering that paleopolyploid species, such as soybean, have larger repertoires of PKs. Indeed, because soybean’s duplication events occurred much earlier than sugarcane’s (having occurred ∼13–59 MYA; Schmutz et al., 2010), its homologous chromosomes are not treated as allelic copies. Therefore, it is only natural that more PK genes were identified in the two kinomes compiled for Sbi, namely, 2,099 (Lehti-Shiu and Shiu, 2012) and 2,166 (Liu et al., 2015) PKs, whereas Ssp which underwent very recent WGDs, contained many fewer PK genes when allelic copies were considered.

In Sbi, PK genes were more commonly located in subtelomeric regions. This pattern was even more evident when only tandemly duplicated PKs were considered; similar (though less pronounced) patterns were observed in the kinomes of soybean (Liu et al., 2015), *T. aestivum* (Yan et al., 2017), *Gossypium raimondii*, and *Gossypium barbadense* (Yan et al., 2018). Yan et al. (2017) noted that this pattern is consistent with the higher gene and expressed sequence tag densities in distal regions of chromosomes of *T. aestivum* and inferred that such a location pattern could indicate chromosomal rearrangements. Our findings are equally compatible with the genomic landscape of sorghum: in this species, the density of genes – especially paralogs – is markedly higher in chromosome extremities, whereas pericentromeric regions are very rich in long terminal repeat retrotransposons (Paterson et al., 2009; Mace and Jordan, 2011). The gene density in Ssp on the other hand, is less skewed toward subtelomeric regions (Zhang et al., 2018), which might explain why we did not observe such a clear pattern for PK gene distribution in the species. An analogous observation was made by Zhang et al. (2019a) when comparing the genomic organization of Sbi, *S. officinarum*, and *S. robustum*. These authors note that, despite observing considerable collinearity between species, genes that were widely dispersed in *Saccharum* linkage groups were much more tightly clustered in subtelomeric regions on Sbi chromosomes. The same pattern is evident in the synteny plot between the Sbi and Ssp kinomes (Figure 5D): While many Sbi PK genes are present in Ssp they are much more widely distributed along chromosomes in Ssp. The dispersion of Ssp kinase genes between chromosomes and allelic copies was also relatively balanced and somewhat proportional to chromosomal length. Overall, this finding is similar to the patterns of kinase genes obtained for rice (Dardick et al., 2007), pineapple, and grapevine (Zhu et al., 2018a,b), even though genes are more unevenly distributed along chromosomes.

Differences in PK composition may lead to different functional profiles. Similarly, structural divergences may arise at distinct points in evolutionary history (Teich et al., 2007; Liu et al., 2015), contributing to different domain organizations and, subsequently, to diverse functions (Xu et al., 2012). Although PKs in the same subfamily have similar intron distribution profiles in wheat (Yan et al., 2017), several compositional differences were detected in the soybean kinome (Liu et al., 2015); we also detected such differences in Ssp and Sbi. In the Sbi kinome, the distribution of introns across subfamilies was more organized than that in the Ssp kinome, i.e., the distribution of introns in Ssp was more variable among the PKs subfamilies. This finding indicates the more recent intron/exon reorganization of Ssp PKs and that gene reorganization might have occurred after these species diverged, a hypothesis that can be investigated for a deeper understanding of PKs evolution in Saccharinae. This data also benefits further comparisons among subfamilies and gene evolution within them. The NEK, CK1_CK1-Pl, PEK_GCN2, and TKL_CTR1-DRK-2 families had the most prominent structural organization in both Sbi and Ssp. All of these families play essential roles in cellular processes (Moniz et al., 2011; Tan and Xue, 2014; Takatani et al., 2015; Varberg et al., 2018; Karpov et al., 2019; Pei et al., 2019), which requires a higher level of organization. In contrast with the highly organized gene profile, Ssp PK subfamilies had the largest number of domains, corroborating the most recent possible gene organization of PKs.

The number of potential PK genes with a domain coverage less than 50% can indicate that they represent atypical kinases or pseudogenes (Lehti-Shiu and Shiu, 2012; Liu et al., 2015). For Sbi, this criterion resulted in the exclusion of ∼3% of sequences (57 genes) with significant correspondences with PKs. In Ssp however, almost 20% of the initially identified PKs were discarded (735 genes). Lehti-Shiu and Shiu (2012) found that 9.6% of all kinases initially identified in 25 species exhibited a domain coverage of less than 50%, and this value varied considerably in later studies that employed the same methodology. We can also speculate regarding the influence of polyploidization on the pseudogenization of PK genes. Although no kinomes have been published for other autopolyploid species, similar findings were reported in allopolyploids. The kinome of the allohexaploid *T. aestivum* contains ∼22% atypical kinases, whereas the kinomes of two of its diploid parental species, *Triticum urartu* and *Aegilops tauschii*, contain ∼16 and ∼14% atypical kinases, respectively. Similarly, the kinomes of *G. raimondii* and *Gossypium arboretum* contain ∼4 and ∼9% atypical kinases, respectively, whereas in the kinomes of the allotetraploids *Gossypium hirsutum* and *G. barbadense*, ∼12% of PKs have such characteristics. The larger numbers of kinase genes with atypical domains in polyploid genomes might have resulted from more frequent pseudogenization events in these species and subsequent WGD, a long-proposed consequence of gene duplication and thus of polyploidization (Magadum et al., 2013).

Overall, the Ssp and Sbi kinomes exhibited similar duplication patterns; in both species, the most common type of PK duplication was segmental duplications, followed by tandem duplications. These duplication events are usually reported as the two main contributors to PK expansion in the genomes of several other species, especially in the RLK-Pelle superfamily (Champion et al., 2004; Dardick et al., 2007; Wei et al., 2014; Liu et al., 2015; Dezhsetan, 2017; Zhu et al., 2018a,b). Gene retention by tandem duplication in kinases has already been identified, with very high rates in several plants (Lehti-Shiu and Shiu, 2012), and considerable correlation with different kinds of stress (Freeling, 2009). The association of PK expansion through such events with defense response and signaling pathways has been widely reported in kinome studies (Zulawski et al., 2014; Liu et al., 2015; Yan et al., 2018; Zhu et al., 2018a,b), with these events being more pronounced in the RLK-Pelle group. In the Ssp and Sbi kinomes, we found several subfamilies in this group with tandem duplications (mostly in LRR families). By analyzing GO biological process categories related to these events (Figure 3), we found a considerable frequency of categories related to the defense response; however, other general categories were also frequent, which is explained by the numerous processes related to these subfamilies. Interestingly, in RKF-3 (in the RLK-Pelle group) in Ssp all duplications were associated with tandem events, and members of this family have already been linked to stress responses and extracellular signaling (Huang et al., 2014; Vaid et al., 2016). Even with this high similarity, several differences in the distribution of tandemly organized genes within subfamilies were found between the Ssp and Sbi kinomes. These species- and chromosomal region-specific organizational characteristics were previously noted by Yan et al. (2018) in a comparison of cotton kinomes. With respect to genome organization in Ssp and Sbi, different forms of tandem events have already been found (Wang et al., 2010), with specific gene organization patterns within each genome (Zhang et al., 2018).

Segmental duplication events were also the major contributors to PK expansion in other species; in the soybean kinome, these events accounted for the origin of more than 70% of the PKs (Liu et al., 2015); in grapevine, they were estimated to be responsible for the origin of ∼30% of the kinases and were thought to be especially relevant in the expansion of the RLK-Pelle family (Zhu et al., 2018b). The most striking duplication-related difference between the Ssp and Sbi kinomes was the distribution of the rate of nonsynonymous mutations (*Ks*), which was used to estimate the time of occurrence of segmental duplications. While the range of Sbi PK *Ks* values was comparatively wide, peaking at 0.65–0.85 (Supplementary Figure 6B), the *Ks* values of Ssp exhibited a very prominent peak between 0 and 0.05 range; additionally, the further distribution of *Ks* was somewhat similar to that in Sbi (Supplementary Figure 6A). Based on the clock-like rates of synonymous substitutions, we estimated that the time of occurrence of this large number of segmental duplications with *Ks* < 0.05 was less than 3.8 MYA. Thus, we postulate that the *Ks* distribution in Ssp is a consequence of the recent polyploidization events in sugarcane; this hypothesis is supported by recent indications that the *Saccharum*-specific WGDs occurred in the last 3.1–4.6 million years (Kim et al., 2014; Zhang et al., 2018). This is further reinforced by the findings reported in *Gossypium* spp. kinomes; a profile of *Ks* distributions very similar to that in Ssp was observed in the allotetraploids *G. hirsutum* and *G. barbadense* but not in its diploid relatives (Yan et al., 2018), strengthening the connection of this profile to WGD events.

### Sugarcane and Sorghum Kinomes’ Expression Patterns

Several RNA-Seq experiments were used to estimate the expression patterns of kinase subfamilies across a considerable range of tissues and genotypes. Due to the similar expression patterns within subfamilies (Liu et al., 2015) and the possibility of detecting clearer expression patterns in different subfamilies than at the individual gene level, expression analysis was performed, combining expression levels of genes from subfamilies, instead of individual genes. The differences among samples were more evident when separated by tissue instead of genotype and species (Figure 7), possibly because of the strong conservation of PKs and their importance in several fundamental biological processes. In addition, as Liu et al. (2015) suggested, we also recognized that the Ssp and Sbi kinomes’ expression is shaped by the physiological characteristics of these species. The highest expression levels were found for members of the CMGC group in both the Sbi and Ssp kinomes. Additionally, in the AGC, CAMK, and CK1 groups, we found high expression levels in several subfamilies. These findings were previously reported in other plants, suggesting an association of these groups with developmental processes (Liu et al., 2015; Zhu et al., 2018a,b). Interestingly, even though RLK-Pelle subfamilies account for the largest number of PKs among the kinomes, they were not among the top overexpressed subfamilies.

Despite having only one kinase member in Ssp and Sbi, the AGC_PKA-PKG subfamily showed one of the highest average expression values across Ssp tissues. In addition to the unremarkable expression of RLK-Pelle members, the high AGC_PKA-PKG expression corroborates the observation that the expression in the Ssp and Sbi kinomes was not related to the number of family members across families and groups. If we assume that PK subfamilies might have increased in size through duplication events, this might be a case of dosage balance, a phenomenon in which the function of regulatory genes is sensitive to a stoichiometric equilibrium (Birchler and Veitia, 2014). Thus, PK families composed of more members (which survived duplications and thus present more copies) have a tendency toward lower average expression, as has been demonstrated in other plants (Birchler and Veitia, 2012). Additionally, AGC_PKA-PKG subfamily has been reported as broadly important in both Ssp and Sbi. In Ssp studies have demonstrated the association of its members with signaling pathways (Kasirajan et al., 2020), cell proliferation (Li et al., 2016b), infection responses (Santa Brigida et al., 2016; Xu et al., 2018), hormone signal transduction in response to drought (Li et al., 2016a), and pathways related to sucrose storage and photosynthesis (Hoang et al., 2017; Thirugnanasambandam et al., 2017). In Sbi, the importance of this family is also linked with stress and signal responses (Li et al., 2018; Parra-Londono et al., 2018; Nagaraju et al., 2020; Vikal et al., 2020). Therefore, these insights into expression patterns constitute a valuable reservoir of information for analyzing the importance of Ssp and Sbi kinases.

The final analysis performed using RNA-Seq aimed to establish closer relationships among kinase subfamilies in Sbi and Ssp through coexpression networks, enabling biological inferences using connection patterns. The gene coexpression networks were constructed with pairwise correlations (similarity scores) from the gene expression quantification data (Serin et al., 2016). Pearson correlation coefficients were used because of their reasonable performance in RNA-Seq datasets (Ballouz et al., 2015). Moreover, as Liu et al. (2015) suggested, we constructed the networks based on subfamily relationships instead of single genes because of the enhanced functional interpretability and general inferences allowed by this approach. Complex networks have been widely applied to visualize complex biological systems (Barabási, 2016), and constitute a powerful tool for modeling gene interactions (Zhao et al., 2010). For kinase subfamily representations, these networks can facilitate the interpretation of relevant relationships among sets of kinases and provide insights into the interactions among metabolic mechanisms. Such applications are possible because similar expression patterns on genes belonging to the same pathways reflect the network structure (Lee et al., 2015), thus providing a tool to model these complex molecular interactions (Ficklin and Feltus, 2011).

Together with the network representations, we used community detection methodologies to identify modules of cohesive elements, which possibly indicate more strongly interconnected metabolic relationships (Mitra et al., 2013; Mall et al., 2017; Zhang and Yin, 2020). This structural organization constitutes a reservoir of genetic information among kinomes and provides important insights into how PK subfamilies biologically interact. When some subfamilies without a significant amount of relationships with other elements were excluded [nine Sbi subfamilies in communities 3, 4, and 6–10 (Supplementary Table 40) and 13 Ssp subfamilies in communities 3–9, 11–13, and 15–17 (Supplementary Table 41)], the network structures (Figure 8) indicated that all of the subfamilies were interconnected, considering the nonrandom dependencies across subfamilies captured by the established correlation coefficient threshold (Ficklin and Feltus, 2011). Even though they have specific functions, all kinase subfamilies play roles in several common metabolic processes, and this commonality is clearly reflected in the network structures.

In addition, considering the roles of PKs in metabolic signaling and stress responses, the organization of several subfamilies is reasonably conserved among different plant species (Lehti-Shiu and Shiu, 2012). By comparing the Sbi and Ssp networks, we identified a substantial core of similarity between the subfamily interactions in these species, which might indicate several analogous expression profiles (Figure 7). In addition to the comparison of network connectivities, other topological characteristics were used to identify important features in the organization of kinome subfamilies. Hub and betweenness measures were calculated to supply evidence regarding how specific subfamilies are important in most metabolic processes involving kinases.

Within a network structure, elements with the most connections are called hubs (Barabási, 2016). These nodes have been used to identify functionally critical components and as an additional approach to describe the network structure (Hong et al., 2013; Azuaje, 2014; van Dam et al., 2018). In the constructed networks, the hub nodes indicate kinase subfamilies with the most correlations, which might represent sets of kinases with influential roles in diverse metabolic mechanisms in kinomes. Interestingly, the Sbi and Ssp networks did not exhibit high overlap of hub nodes. This observation provides evidence that although there are several similarities among the kinase expression profiles in the species, and the same biological cascades are activated, as indicated by the GO analyses (Supplementary Figure 5), the mechanism by which the expression balance is achieved is species-specific. In fact, previous studies in polyploids have shown that this balancing varies even among lines of the same species (Mutti et al., 2017).

In both Sbi and Ssp networks and those constructed in other kinome studies (Liu et al., 2015; Zhu et al., 2018b), different members of RLK-Pelle (mostly those in the LRR and RLCK families) were identified as hubs. Considering the described abundance of these families, their tandem duplications, and related functional implications, the strong influence of such nodes on the correlations among kinase subfamilies was expected. CMGC group subfamilies were also identified as hub elements, as observed in the soybean kinome (Liu et al., 2015). In the sugarcane network, the GSK and CDK families had a considerable number of connections, which is clearly explained by the very high number of pathways in which their members are involved, as previously noted. Additionally, CDK has already been found to be related to stress signaling in Ssp (Patade et al., 2011) and Sbi (Challa and Neelapu, 2018). In Sbi, DYRK also had a high node degree. Interestingly, members of this family have already been found to be related to the suppression of photosynthesis activity (Kimura and Ishikawa, 2018); thus, the importance and impact of this family among kinases is evident. Among the other hubs, the STE group (STE20 family) was also important in the Ssp and Sbi networks, which can be explained by the high number of biological cascades related to this subfamily (Xiong et al., 2016).

Several factors can explain why a subfamily constitutes a hub in our constructed networks, such as a high expression level (CMGC_GSK, CAMK_OST1L, CK1_CK1, and CMGC_DYRK-YAK), a large number of subfamily members (RLK-Pelle_LRR-III), the occurrence of tandem duplications (IRE1), a more structured gene organization considering intron-exon structures (RLK-Pelle_RLCK-XII-1, RLK-Pelle_LRR-VI-1, and CMGC_GSK), and the presence of diverse functional domains (RLK-Pelle_LRR-III, CMGC_CDK-CRK7-CDK9, and RLK-Pelle_LRR-VII-1) or multikinase domains (AGC_RSK-2, RLK-Pelle_LRR-III, and CMGC_CDK-CRK7-CDK9). However, we did not observe a consistent feature profile required for a subfamily to be considered a hub. Evidence supports the hubs’ importance; however, the real reasons for their key importance within these structures are likely to be linked with functional properties, as widely discussed in other coexpression studies (Goel et al., 2018; Tai et al., 2018; Wang et al., 2018; Zou et al., 2019; Ding et al., 2020).

In addition to hub descriptions, edge betweenness measures also have high interpretability considering the complex system modeled by the networks. These calculations are based on properties from the entire network (Dunn et al., 2005), exploiting the network flow and identifying possible essential interactions for the visual configuration (van Dam et al., 2018). In both the Sbi and Ssp networks, a clearly separated group of PKs that was connected with the other elements by only one or a few connections was observed. This network configuration might indicate important relationships among kinase subfamilies, providing evidence indicating how these specific subfamilies can interconnect. In Ssp the most critical connections identified by betweenness calculations were found in the RLK-Pelle_L-LEC/RLK-Pelle_LRR-VIII-1 and RLK-Pelle_CR4L/RLK-Pelle_LRR-Xb-1 subfamilies. These nodes are members of families with undeniable importance, as seen in the network structure. The bridges in these kinase-kinase interactions can be explained by the large number of members that can act in a connected manner, which is less evident in other subfamilies. However, as observed in the hub configurations, these structures are more evidently linked with functional roles, such as interconnected signaling pathways.

In the Sbi network, on the other hand, the highest betweenness values were found in the CAMK_CDPK/RLK-Pelle_LRR-VI-2 and CAMK_CDPK/CMGC_RCK subfamilies. Interestingly, CAMK_CDPK genes have been extensively indicated to be located at important genomic regions regulating growth, development, and resistance mechanisms to several types of abiotic and biotic stresses in both Sbi (Pestenácz and Erdei, 1996; Nhiri et al., 1998; Jain et al., 2008; Li et al., 2010; Monreal et al., 2013; Usha Kiranmayee et al., 2017) and Ssp (Li et al., 2016b; Marquardt et al., 2017; Ling et al., 2018; Dharshini et al., 2020; Srivastava and Kumar, 2020; Su et al., 2020b), further supporting the association of functional characteristics in the network structure.

## Conclusion

This study provided an extensive reservoir of genetic and molecular information for both Sbi and Ssp. Considering the incontestable importance of kinases in several essential biological processes, the identification, categorization, and analysis of the kinomes of these species resulted in an important compendium of knowledge for use in further studies. Clear similarities were found in protein properties, domain compositions, genomic organization, expression profiles, and subfamily interactions. However, we also observed pronounced differences in duplication events, which probably arose from Ssp recent WGDs, facilitating understanding of how the Sbi and Ssp kinomes have evolved considering this vast protein superfamily. Through coexpression networks we could supply insights into kinase subfamilies’ interactions in Sbi and Spp; we could define a common interactional structure, but observed substantial differences on the subfamilies’ communications, quantified with community structures, hub, and betweenness scores. Further assessments of elements with relevant influence over the network architecture could highlight subfamilies with direct influence over the cascade of kinase cellular mechanisms. More detailed studies on these groups should contribute to the understanding of molecular signaling and stress responses in Sbi and Ssp.

## Data Availability Statement

The datasets presented in this study can be found in online repositories. The names of the repositories and accession numbers can be found at: National Center for Biotechnology Information (NCBI) GenBank, https://www.ncbi.nlm.nih.gov/genbank/, PRJEB38368, PRJNA681593, PRJEB41560 and PRJEB40481.

## Author Contributions

AA and RP performed all analyses and wrote the manuscript. AG, FC, and GH assisted in processing the sugarcane RNA-Seq data, and together with CC-S, MM, and DS, were responsible for the sugarcane RNA-Seq experiments. MMC assisted in the functional analyses of the kinase subfamilies. LS contributed to the identification of the kinases. JN assisted in the kinase categorization. LP, ML, MSC, and TB were responsible for the sugarcane field experiments. MQ assisted in the network analysis. WP assisted in the pipeline definition and kinase categorization. GM and AS conceived the project. All authors contributed to the article and approved the submitted version.

### Conflict of Interest

The authors declare that the research was conducted in the absence of any commercial or financial relationships that could be construed as a potential conflict of interest.

## Figures and Tables

**Table:** 

Aco	*Aquilegia coerulea*
AGC	Cyclic AMP-dependent protein kinase (cAPK), cGMP-dependent protein kinase, and lipid signaling kinase families
Aly	*Arabidopsis lyrata*
Ath	*Arabidopsis thaliana*
B-lectin	D-mannose-binding lectin
Bdi	*Brachypodium distachyon*
CAMK	Calcium- and calmodulin-regulated kinase
Ccl	*Citrus clementina*
CDS	DNA coding sequence
CK1	Casein kinase 1
CMGC	Cyclin-dependent kinase, mitogen-activated protein kinase, glycogen synthase kinase, and cyclin-dependent kinase-like kinase
Cpa	*Carica papaya*
Cre	*Chlamydomonas reinhardtii*
Csa	*Cucumis sativus*
Csi	*Citrus sinensis*
DUF26	Domain of Unknown Function 26
Egr	*Eucalyptus grandis*
ER	Endoplasmic reticulum
GM	Gene model
Gma	*Glycine max*
GO	Gene Ontology
GUB	Galacturonan-binding
HMM	Hidden Markov model
IRE1	Inositol-requiring kinase 1
*Ka*	Nonsynonymous substitution rates
*Ks*	Synonymous substitution rates
LRR	Leucine-rich repeat
LRRNT	Leucine-rich repeat N-terminal domain
Mes	*Manihot esculenta*
Mgu	*Mimulus guttatus*
Mtr	*Medicago truncatula*
MW	Molecular weight
MYA	Million years ago
Osa	*Oryza sativa*
PEK	Pancreatic eukaryotic initiation factor-2alpha kinase
PK	Protein kinase
pI	Isoelectric point
Ppa	*Physcomitrella patens*
Ppe	*Prunus persica*
Ptr	*Populus trichocarpa*
Rco	*Ricinus communis*
RLK	Receptor-like kinase
S-locus-glycop	S-locus glycoprotein
Sbi	*Sorghum bicolor*
Sit	*Setaria italica*
Smo	*Selaginella moellendorffii*
Ssp	*Saccharum spontaneum*
STE	Serine/threonine kinase
TKL	Tyrosine kinase-like kinase
TPM	Transcripts per million
Vca	*Volvox carteri*
Vvi	*Vitis vinifera*
WAK	Wall-associated receptor kinase
WGD	Whole-genome duplication
Zma	*Zea mays*

## Supplementary Material

The Supplementary Figures and Tables for this article have been deposited in Figshare (https://figshare.com/): https://doi.org/10.6084/m9.figshare.c.5122460.v2. The Supplementary Results section can be found online at: https://www.frontiersin.org/articles/10.3389/fpls.2021.668623/full#supplementary-material
