# Supplementary material for: The Wild Sugarcane and Sorghum Kinomes: Insights Into Expansion, Diversification, and Expression Patterns
Source: Front Plant Sci. 2021 Jul 7;12:668623. doi: 10.3389/fpls.2021.668623 (PMC8294386; doi:10.3389/fpls.2021.668623)
Supplement: Supplementary file 1 [file Data_Sheet_1.PDF]

Supplementary Material

1 Supplementary Results

1.1. Genome-wide Identification of PKs in Sugarcane and Sorghum

All *Saccharum spontaneum* (Ssp) and *Sorghum bicolor* (Sbi) protein sequences available were aligned against kinase domains using HMMER, and 3,729 (Ssp) and 1,910 (Sbi) different sequences showed significant correspondences. To avoid redundancies, we removed Sbi isoforms, and we discarded 810 (Ssp) and 66 (Sbi) sequences with less than 50% of minimum domain coverage to avoid atypical kinases or pseudogenes (Lehti-Shiu and Shiu, 2012). Ultimately, we identified 2,919 putative Ssp and 1,210 putative Sbi protein kinases (PKs) (Supplementary Tables 3 and 4). The data indicate that some PKs (228 Ssp and 49 Sbi PKs) contained multiple kinase domains.

The genome-wide identification of Ssp PKs was performed without prior knowledge of allelic relationships among genes; however, due to the allele specificity of the Ssp genome, we also evaluated their gene model (GM) organization, in which different allele copies and paralog and tandem duplications are associated with one representative GM. The 2,919 Ssp PKs corresponded to 1,345 different GMs (~5% higher than that obtained with Sbi PKs). By analyzing the GM description file, we identified 3,717 different gene configurations for these 1,345 GMs, which exceeded the number of kinases initially detected (2,919). We verified that this numerical divergence was related only to the presence of tandem and paralogous duplications, which were not classified as PKs with the original identification methodology. When the number of PKs identified solely from different allele copies was verified, 2,575 PKs were identified both by the original identification method and when checking the GM file.

PKs were further classified into groups and subfamilies according to the top-scoring correspondence to hidden Markov models (HMMs) of 25 plant species (Lehti-Shiu and Shiu, 2012). This process resulted in the identification of 119 and 120 kinase subfamilies in Ssp and Sbi, respectively (Supplementary Tables 5 and 6), corresponding to 20 different groups. This classification was confirmed by phylogenetic trees (Supplementary Figures 1-3) estimated based on Sbi PKs, Ssp PKs and all PKs from the two species. In the dendrogram, only seven sequences in Ssp and two sequences in Sbi did not cluster with any other subfamily; instead, these were included in an "Unknown" category and considered probable novel gene kinase subfamilies. The comparison of Ssp GMs and Sbi PKs revealed that the number and relative proportion of proteins in each group were similar (Supplementary Table 7), and the same values were obtained for 40% of the subfamilies.

The comparison of the Sbi and Ssp kinomes alone reveals that their subfamily composition profiles are very similar. The only subfamily found exclusively in Sbi was the pancreatic eukaryotic initiation factor-2alpha kinase (PEK_PEK). Although PEKs are responsible for the phosphorylation of eukaryotic translation initiation factor 2 subunit alpha (eIF2α) (Immanuel et al., 2012), each subfamily is involved in the response to different stresses (Donnelly et al., 2013). The PEK_GCN2 subfamily was found in both species, and its activation is related to amino acid and glucose deprivation (Yang et al., 2000; Deval et al., 2009; Baker et al., 2012), viral infection (Berlanga et al., 2006; Krishnamoorthy et al., 2008) and UV irradiation (Grallert and Boye, 2007). PEK_PEK kinases

are particularly activated during endoplasmic reticulum (ER) stress (Baker et al., 2012) and are homologous to inositol-requiring kinase 1 (IRE1) proteins (Urano et al., 2000), which are also activated in response to ER stress (Liu et al., 2007a) and were found in both the Sbi and Ssp kinomes.

The most abundant group in both species was the receptor-like kinase (RLK)-Pelle group, which accounted for ∼70% of the PKs with leucine-rich repeat (LRR), RLCK, DLSV, L-LEC and SD-2b subfamilies having the most pronounced compositions. In the cotton kinome, for instance, LRR subfamilies have been suggested to be significantly associated with growth, development and defense responses (Yan et al., 2018). In Sbi, the LRR family has broadly been linked with the response to several types of stress (Kawahigashi et al., 2011; Azzouz-Olden et al., 2020; Filiz and Kurt, 2020), playing roles related to signal transduction in response to extracellular signals (Azzouz-Olden et al., 2020; Dhaka et al., 2020; Vikal et al., 2020), pollen development (Dhaka et al., 2020), metabolism, and chaperone functions (Vikal et al., 2020). In Ssp, this family has been associated with defense response processes (Xu et al., 2018; Yang et al., 2019), hormone metabolism (Chen et al., 2019), cellulose and lignin biosynthesis (Kasirajan et al., 2018), and sucrose synthesis (Vicentini et al., 2009). In addition to the remarkably important LRR family, the RLCK, DLSV, L-LEC, and SD-2b families are also involved in diverse essential mechanisms. Because RLCK members do not contain extracellular and transmembrane domains (Gao and Xue, 2012; Zulawski et al., 2014), these proteins are generally involved in more specific processes (Jurca et al., 2008). In addition to disease resistance, RLCKs have been shown to be related to growth, immune responses (Yan et al., 2018; Zhu et al., 2018a), and vegetative development (Jurca et al., 2008; Gao and Xue, 2012). Together with RLCK family members, DLSV members were found to be differentially expressed in soybean tissues in stress experiments (Liu et al., 2015). The DLSV family includes Domain of Unknown Function 26 (DUF26), SD-1, LRR-VIII, and VMA-like proteins (Lehti-Shiu and Shiu, 2012), which mediate the control of stress responses and development (Vinagre et al., 2006; Vaattovaara et al., 2019), with some members being associated with signaling pathways regulating the responses to cold and infection (Yan et al., 2017). L-LEC and SD-2b have established associations with the defense responses (Chen et al., 2006; Wei et al., 2014) and with stomatal immunity regulation via an L-LEC member (Desclos-Theveniau et al., 2012) and with self-incompatibility via SD-2b (Stein et al., 1991). The essentiality of mechanisms shared by these families indicates their functional importance among plants (Vaattovaara et al., 2019) and demonstrates their importance in the expansion and maintenance of the Sbi and Ssp kinomes.

1.2. Characterization of PKs

Ssp and Sbi PKs were distributed across all Ssp and Sbi chromosomes (Figures 2A and 2B). Sbi PK quantities ranged from 67 (5.54%) on chromosome 7 to 184 (15.21%) on chromosome 3 (Supplementary Table 8). In Ssp (Supplementary Table 9), quantities across haplotypes were similar, and in all of them, chromosomes 2 and 6 had the most and fewest PKs, respectively. The accumulation of PKs generally increased with increases in chromosomal length.

The intron distribution differed between Ssp and Sbi PKs (Supplementary Tables 10 and 11) and did not exhibit a clear distribution pattern on a specific chromosome (Figures 2A and 2B). A large number of PKs were intronless (154 in Sbi and 329 in Ssp). Additionally, all identified PKs were analyzed against the Pfam database to retrieve related nonkinase domains. In Sbi, we identified 70 additional domains (Supplementary Table 12) distributed across 662 PKs (Supplementary Table 13). Interestingly, none of these additional domains were found in the 49 PKs containing multiple kinase domains (Supplementary Table 14). In Ssp, we identified 168 additional domains (Supplementary

Table 15) across 1,423 PKs (Supplementary Table 16). The 228 Ssp PKs with multiple kinase domains (Supplementary Table 17) also did not present nonkinase domains. These additional domains were similar in Sbi and Ssp PKs (60 domains in common).

A full Gene Ontology (GO) annotation of Sbi and Ssp PKs was performed with Blast2GO (Supplementary Tables 18 and 19). In Sbi, we found 1,581 different GO terms related to 18,320 correspondences among the PKs. These terms were separated into 3,857 (21.05%) terms related to the cellular component GO category, 3,752 (20.48%) to the molecular function category, and 10,711 (58.47%) to the biological process category. In Ssp, we found more categories (1,875) and more correspondences (44,582) due to the larger size of the Ssp kinome. However, the proportion of GO terms was similar: 9,193 (20.62%) in the cellular component category, 9,429 (21.15%) in the molecular function category, and 25,960 (58.23%) in the biological process category. This clear similarity can be observed in pie charts in Figures 2C and 2D. Using REViGO software, treemaps were generated to summarize the GO categories related to biological processes (Supplementary Figures 5A and 5B); the most abundant biological processes were related to protein phosphorylation, defense response and cellular development.

The annotation of PKs based on GO terms corroborated the accuracy of their identification. For instance, in both the Ssp and Sbi kinomes, the five most frequently appearing annotated GO terms were (I) defense response to oomycetes, (II) protein serine/threonine phosphorylation, (III) binding, (IV) plasma membrane and (V) pollen development (Figures 2C and 2D). Indeed, terms (II) and (III) exhibit the most obvious associations, as PKs catalyze the phosphorylation of proteins by transferring terminal phosphate groups from ATP to serine, threonine or tyrosine residues in other proteins—a process that involves the binding of PKs to their targets (Hunter, 1995). A large portion of eukaryotic plant kinases are grouped into the RLK superfamily and are thus located in the plasma membrane, which explains term (IV). Additionally, PKs have been frequently shown to participate in responses to infection by various oomycetes (Hall et al., 2007; Blanco et al., 2008; Hok et al., 2011, 2014; Carella et al., 2019) and in pollen development (Zhang et al., 2001; Xu et al., 2011; Lafleur et al., 2015; Chen et al., 2016; Li et al., 2018), explaining terms (I) and (V). This finding was maintained when the annotation results were summarized in treemaps (Supplementary Figures 5A and 5B); first, terms associated with protein phosphorylation were strongly represented in the kinomes of both species. This summarization also highlights the broad presence of terms in which plant PKs are widely and historically known to be involved, such as defense responses (Chen et al., 2006; Tena et al., 2011; Wei et al., 2014; Xu et al., 2018; Yang et al., 2019), cellular development (Jin et al., 2002; Matschi et al., 2013; Komis et al., 2018), regulation of stomatal closure (Li et al., 2000; Mustilli et al., 2002; Lee et al., 2016) and development of leaves and pollen (Roe et al., 1993; Benjamins et al., 2001; Khew et al., 2015).

For Sbi PKs, we investigated the possible occurrence of alternative splicing (AS). One hundred Sbi kinase genes were found to undergo associated AS, and a GO analysis of the most frequent biological processes associated with these genes (Supplementary Figure 5C) showed changes in the most frequent categories compared with those obtained when considering the entire dataset of PK-related terms. The most frequent category was defense response, which was the third most frequent in the entire set of Sbi PK GO terms. Additionally, programmed cell death was included as a category instead of cell growth. For this species, we also investigated 100 PKs that are possibly subject to AS, a process that leads to the production of different mRNA isoforms from a single gene, which expands the functional diversity of the gene. AS is extensively reported to regulate plant development, circadian clocks and responses to environmental stimuli, particularly stresses (Filichkin et al., 2015;

Shang et al., 2017). When only alternatively spliced PKs were annotated and summarized (Supplementary Figure 5C), we observed similarities to the categories associated with all GO terms in the two species analyzed. One notable difference was the inclusion of a category that included terms related to programmed cell death, a stress-triggered process (Danon et al., 2000) controlled by PKs (Tang et al., 2005; Liu et al., 2007b; Lachaud et al., 2013; Wrzaczek et al., 2014; Yadeta et al., 2016). A few PKs that function in response to biotic and abiotic stresses have been shown to undergo AS (Rostoks et al., 2004; Koo et al., 2007; Lin et al., 2010), which could explain the high frequency of this category with alternatively spliced PKs.

Additionally, we analyzed the ratio of synonymous to nonsynonymous mutations (Ka/Ks), which is used to determine the type of selection acting on a gene (Zhang et al., 2006). We found that in the two kinomes the large majority of segmentally duplicated PKs were under negative selection (Ka/Ks<1), while a smaller percentage were under positive selection (Ka/Ks>1), and very few were under neutral selection (Ka/Ks=1). This pattern is similar to those observed in the soybean, grapevine and pineapple kinomes (Liu et al., 2015; Zhu et al., 2018a, b) and to those reported in smaller gene families in Ssp (Wang et al., 2019b; Li et al., 2020) and sorghum (Malviya et al., 2016; Anand et al., 2017; Mittal et al., 2017).

We also explored the presence of signal peptides and transmembrane helices in the PKs and investigated their estimated molecular weights (MWs), theoretical isoelectric points (pIs), and subcellular localization (Supplementary Tables 20 and 21). Among the Sbi PKs, ~40% were predicted to contain signal peptides (Figure 2A), whereas ~30% of the Ssp PKs contained these peptides (Figure 2B). Most Ssp PKs (~59%) did not contain transmembrane helices, whereas 50% of Sbi PKs did not (Figures 2C and 2D). The results indicated high divergence among the methods for predicting subcellular localization; thus, we considered only the predictions identified by a consensus of at least two of the three tools used. The localization of 1,425 Ssp and 616 Sbi PKs was predicted to be the chloroplast, cytoplasmic, extracellular, mitochondrial, nuclear or membrane regions (Figures 2C and 2D). The most frequently identified localization was the membrane, as also indicated by the high frequency of the membrane GO term.

Various attributes of PKs—number of introns, pIs, MWs, presence of signal peptides and transmembrane helices, duplications, and domain composition—are summarized at subfamily level in Supplementary Tables 22 (Sbi) and 23 (Ssp). To characterize kinase subfamily gene structures, we first calculated the mean quantity of introns per kinase in each subfamily and then determined the standard deviation and coefficient of variation. Several subfamilies contained only one representative gene (30 in Sbi and 33 in Ssp). In Ssp, some of these subfamilies exhibit high intronic divergences in gene allelic copies (with coefficients of variation ranging from 0 to ~141%). The analysis of only the subfamilies with more than one member revealed increased coefficients of variation (ranging from 0 to ~241%), corresponding to significant discrepancies in the gene organization within kinase subfamilies. By filtering the subfamilies with a maximum coefficient of variation of 20% and at least two PKs, we identified 37 Sbi and 12 Ssp subfamilies with a more cohesive structure, but most included only a few PKs.

The protein properties across kinase subfamilies did not exhibit considerable differences. Based on a maximum coefficient of variation of 20%, 13 and 15 subfamilies in Sbi and Ssp, respectively, presented considerable variations in the pI. The MW exhibited higher variability in Ssp than in Sbi (66 subfamilies with more diverse values, in contrast to 20 in Sbi). Regarding the presence of signal

peptides, all PKs in 18 Sbi PK subfamilies contained these subsequences; the subfamilies with the most members were RLK-Pelle_LRR-V (12 members) and RLK-Pelle_WAK_LRK10L-1 (7 members). In Ssp, all PKs in only eight subfamilies contained signal peptides, with the inositol-requiring kinase 1 (IRE1) and RLK-Pelle_RLCK-X subfamilies each containing five members. Similarly, these highlighted subfamilies also contained transmembrane helices across their proteins.

We also performed several *in silico* analyses to evaluate the molecular characteristics of the PKs identified in the two species. As reported for grapevine (Zhu et al., 2018b), the pIs and MWs of the PKs were generally similar within subfamilies in Ssp and Sbi; these results were expected, as these properties are estimated based on the protein sequence. We observed, however, that Ssp contained many more PK subfamilies with significant variation in the MW than did Sbi, possibly indicating a broader diversity of kinases in Ssp. After verifying the presence of signal peptides in the PK sequences, we estimated that ∼40% of Sbi kinases contained signal peptides, in contrast with ∼30% in Ssp. This percentage is very similar to that in maize, where ∼30% of PKs contain these signal sequences (Wei et al., 2014). Regarding the subcellular localization of the PKs, we noted high divergence in the results obtained with the three machine learning-based methods (Yu et al., 2006; Horton et al., 2007; Sperschneider et al., 2017), which have unique advantages. Therefore, the discordant localizations may not be reliable, and we decided to use a consensus approach, considering only the results consistent between at least two of the tools. Although this process did not allow the subcellular localization of all PKs to be estimated, it did allow us to determine a more consistent predictive set for categorizing the Sbi and Ssp kinomes.

To complement the protein properties observed in kinase subfamilies, the domain composition was described (Supplementary Tables 24 (Sbi) and 25 (Ssp)). Interestingly, the AGC_RSK-2 subfamily had the highest number of PKs with multiple kinase domains in both Sbi and Ssp. Furthermore, we investigated the percentage of multikinase domain-containing proteins among the PKs in each subfamily (Supplementary Tables 22 and 23). The highest percentage (100%) was observed in the AGC_NDR and CMGC_SRPK subfamilies in Sbi and in the CMGC_SRPK, CMGC_CDK-CCRK subfamilies in Ssp. Although the AGC_NDR subfamily did not contain all of the proteins with multiple kinase domains in Ssp, 10 of the 15 (∼66%) had this characteristic. The RLK-Pelle subfamilies, which putatively participate in a wide variety of induced biological processes, showed the largest differences in the domain composition in both Sbi and Ssp, as expected due to its large size. In addition to RLK members, the CMGC_CDK-CRK7-CDK9 (in Ssp) and CMGC_GSK subfamilies were among the top 10% of subfamilies with the largest number of different domains. Therefore, this domain diversity might be explained by the diverse functions performed by these proteins. The CMGC_CDK family (Joubès et al., 2000) integrates several functions of transcription and cell division (Malumbres, 2014). Specifically, the CRK7 and CDK9 subfamilies are related to cell cycle control (Goldberg et al., 2006). Additionally, the GSK subfamily affects numerous signaling pathways (Wrzaczek et al., 2007).

Our study is the first to categorize a kinase superfamily considering allele copies. Although the presence of kinase domains in Ssp PKs was highly conserved, differences in intron exon organization and domain composition were found. The most common compositional differences were related to domain distribution along the allele copies (e.g., inversion of LRR and kinase domains along the sequences), insertion or loss of domains in allele copies (e.g., LRR, antifungal, and uroporphyrinogen decarboxylase domains, as well as domains of unknown function); and duplication of domains (e.g., LRR, legume lectin, EF-hand, and kinase domains). We investigated the number of kinase copies among the alleles of kinases, revealing diversity among the subfamilies (Fig. 6). Although there is no

evidence that the number of allele copies of a gene is related to its expression levels in sugarcane, minor differences across allele copies were observed in Dof genes (Cai et al, 2020). Therefore, our findings suggest specific rearrangements of kinase sequences, indicating possible functional associations. In addition, we verified that not all the PK subfamilies had copies in the four alleles.

Other studied protein families in Ssp also had different pattern distributions across allele copies (Huang et al., 2020; Li et al., 2020). In some studies, the gene structure has been reported to be similar across these copies; however, this pattern is not universal (Ma et al., 2019; Shi et al., 2019). The genomic structure and organization of sugarcane is considerably complex (Sforça et al., 2019), and the pattern of gene distribution across alleles is unclear. Comparing the results of different gene families, there is a variability regarding the number of alleles of each gene (Li et al., 2020; Wang et al., 2019a). Indeed, we observed that the distribution among alleles is also variable for PK subfamilies' genes.Ssp contained more multikinase domains than Sbi, and more repetitions were found in some of its PKs. Similar to soybean and grapevine (Liu et al., 2015; Zhu et al., 2018b), the Sbi kinome contained PKs with only two or three kinase domains, whereas the Ssp kinome contained PKs with two to five kinase domains. Interestingly, the AGC_RSK-2 subfamily was found to have the largest number of multikinase domains in both Sbi and Ssp, accounting for a very high percentage of members of this subfamily, which is explained by the fusion of two PKs in the evolutionary history of the RSK family (Carriere et al., 2008). The AGC_NDR subfamily also exhibited this notable characteristic; however, in this subfamily, the large number of multikinase domains is associated with the insertion of a nuclear localization signal within the kinase domain (Tamaskovic et al., 2003). Moreover, in Sbi and Ssp, the PKs with most kinase domains were in the RLK-Pelle_WAK subfamily, which is functionally linked to cell growth (Gish and Clark, 2011) and whose loss might result in lethality (Wagner and Kohorn, 2001). As the percentage of multikinase domains found in this family was small, we consider that such domains might interact with specific substrates (Liu et al., 2015).

References

Anand, G., Yadav, S., Tanveer, A., Nasim, J., Singh, N. K., Dubey, A. K., et al. (2017). Genome-wide assessment of polygalacturonases-like (PGL) genes of *Medicago truncatula*, *Sorghum bicolor*, *Vitis vinifera* and *Oryza sativa* using comparative genomics approach. Interdiscip. Sci.: Comput. Life Sci. 10, 704-721. doi: 10.1007/s12539-017-0230-y

Azzouz-Olden, F., Hunt, A. G., and Dinkins, R. (2020). Transcriptome analysis of drought-tolerant sorghum genotype SC56 in response to water stress reveals an oxidative stress defense strategy. Mol. Biol. Rep. 47, 3291-3303. doi: 10.1007/s11033-020-05396-5

Baker, B. M., Nargund, A. M., Sun, T., and Haynes, C. M. (2012). Protective coupling of mitochondrial function and protein synthesis via the eIF2α kinase GCN-2. PLoS Genet. 8, e1002760. doi: 10.1371/journal.pgen.1002760

Benjamins, R., Quint, A., Weijers, D., Hooykaas, P., and Offringa, R. (2001). The PINOID protein kinase regulates organ development in Arabidopsis by enhancing polar auxin transport. Development 128, 4057-4067.

Berlanga, J. J., Ventoso, I., Harding, H. P., Deng, J., Ron, D., Sonenberg, N., et al. (2006). Antiviral effect of the mammalian translation initiation factor 2alpha kinase GCN2 against RNA viruses. EMBO J. 25, 1730-1740. doi: 10.1038/sj.emboj.7601073

Blanco, F. A., Zanetti, M. E., and Daleo, G. R. (2008). Calcium-dependent protein kinases are involved in potato signal transduction in response to elicitors from the oomycete phytophthora infestans. J. Phytopathol. 156, 53-61. doi: 10.1111/j.1439-0434.2007.01344.x

Cai M, Lin J, Li Z, Lin Z, Ma Y, et al. (2020). Allele specific expression of Dof genes responding to hormones and abiotic stresses in sugarcane. PloS One 15(1): e0227716. doi:10.1371/journal.pone.0227716

Carella, P., Gogleva, A., Hoey, D. J., Bridgen, A. J., Stolze, S. C., Nakagami, H., et al. (2019). Conserved biochemical defenses underpin host responses to oomycete infection in an early-divergent land plant lineage. Curr. Biol. 29, 2282-2294.e5. doi: 10.1016/j.cub.2019.05.078

Carriere, A., Ray, H., Blenis, J., and Roux, P. P. (2008). The RSK factors of activating the Ras/MAPK signaling cascade. Front. Biosci. 13, 4258-4275. doi: 10.2741/3003

Chen, L., Guan, X., Qin, L., Zou, T., Zhang, Y., Wang, J., et al. (2016). Downregulation of the mitogen-activated protein kinase SlMAPK7 gene results in pollen abortion in tomato. Plant Cell Tissue Organ Cult. 126, 79-92. doi: 10.1007/s11240-016-0979-4

Chen, X., Shang, J., Chen, D., Lei, C., Zou, Y., Zhai, W., et al. (2006). AB-lectin receptor kinase gene conferring rice blast resistance. Plant J. 46, 794-804. doi: 10.1111/j.1365-313X.2006.02739.x

Chen, Z., Qin, C., Wang, M., Liao, F., Liao, Q., Liu, X., et al. (2019). Ethylene-mediated improvement in sucrose accumulation in ripening sugarcane involves increased sink strength. BMC Plant Biol. 19, 285. doi: 10.1186/s12870-019-1882-z

Danon, A., Delorme, V., Mailhac, N., and Gallois, P. (2000). Plant programmed cell death: a common way to die. Plant Physiol. Biochem. 38, 647-655. doi: 10.1016/S0981-9428(00)01178-5

Desclos-Theveniau, M., Arnaud, D., Huang, T. Y., Lin, G. J. C., Chen, W. Y., Lin, Y. C., et al. (2012). The *Arabidopsis* lectin receptor kinase LecRK-V. 5 represses stomatal immunity induced by *Pseudomonas syringae* pv. tomato DC3000. PLoS Pathog. 8, e1002513. doi: 10.1371/journal.ppat.1002513

Deval, C., Chaveroux, C., Maurin, A. C., Cherasse, Y., Parry, L., Carraro, V., et al. (2009). Amino acid limitation regulates the expression of genes involved in several specific biological processes through GCN2-dependent and GCN2-independent pathways. FEBS J. 276, 707-718. doi: 10.1111/j.1742-4658.2008.06818.x

Dhaka, N., Krishnan, K., Kandpal, M., Vashisht, I., Pal, M., Sharma, M. K., et al. (2020). Transcriptional trajectories of anther development provide candidates for engineering male fertility in sorghum. Sci. Rep. 10, 1-16. doi: 10.1038/s41598-020-57717-0

Donnelly, N., Gorman, A. M., Gupta, S., and Samali, A. (2013). The eIF2α kinases: their structures and functions. Cell. Mol. Life Sci. 70, 3493-3511. doi: 10.1007/s00018-012-1252-6

Supplementary Material

Filichkin, S., Priest, H. D., Megraw, M., and Mockler, T. C. (2015). Alternative splicing in plants: directing traffic at the crossroads of adaptation and environmental stress. Curr. Opin. Plant Biol. 24, 125-135. doi: 10.1016/j.pbi.2015.02.008

Filiz, E., and Kurt, F. (2020). Antimicrobial peptides Snakin/GASA gene family in sorghum (*Sorghum bicolor*): genome-wide identification and bioinformatics analyses. Gene Rep. 20, 100766. doi: 10.1016/j.genrep.2020.100766

Gao, L. L., and Xue, H. W. (2012). Global analysis of expression profiles of rice receptor-like kinase genes. Mol. Plant 5, 143-153. doi: 10.1093/mp/ssr062

Gish, L. A., and Clark, S. E. (2011). The RLK/Pelle family of kinases. Plant J. 66, 117-127. doi: 10.1111/j.1365-313X.2011.04518.x

Goldberg, J. M., Manning, G., Liu, A., Fey, P., Pilcher, K. E., Xu, Y., et al. (2006). The Dictyostelium kinome—analysis of the protein kinases from a simple model organism. PLoS Genet. 2, e38. doi: 10.1371/journal.pgen.0020038

Grallert, B., and Boye, E. (2007). The Gcn2 kinase as a cell cycle regulator. Cell Cycle 6, 2768-2772. doi: 10.4161/cc.6.22.4933

Hall, H. C., Samuel, M. A., and Ellis, B. E. (2007). SIPK conditions transcriptional responses unique to either bacterial or oomycete elicitation in tobacco. Mol. Plant Pathol. 8, 581-594. doi: 10.1111/j.1364-3703.2007.00424.x

Hok, S., Allasia, V., Andrio, E., Naessens, E., Ribes, E., Panabières, F., et al. (2014). The receptor kinase IMPAIRED OOMYCETE SUSCEPTIBILITY1 attenuates abscisic acid responses in Arabidopsis. Plant Physiol. 166, 1506-1518. doi: 10.1104/pp.114.248518

Hok, S., Danchin, E. G., Allasia, V., Panabieres, F., Attard, A., and Keller, H. (2011). An *Arabidopsis* (malectin-like) leucine-rich repeat receptor-like kinase contributes to downy mildew disease. Plant Cell Environ. 34, 1944-1957. doi: 10.1111/j.1365-3040.2011.02390.x

Horton, P., Park, K. J., Obayashi, T., Fujita, N., Harada, H., Adams-Collier, C., et al. (2007). WoLF PSORT: protein localization predictor. Nucleic Acids Res. 35, W585-W587. doi: 10.1093/nar/gkm259

Huang, X., Song, X., Chen, R., Zhang, B., Li, C., Liang, Y., et al. (2020). Genome-wide analysis of the DREB subfamily in *Saccharum spontaneum* reveals their functional divergence during cold and drought stresses. Front. Genet. 10, 1326. doi: 10.3389/fgene.2019.01326

Hunter, T. (1995). Protein kinases and phosphatases: the Yin and Yang of protein phosphorylation and signaling. Cell 80, 225-236. doi: 10.1016/0092-8674(95)90405-0

Immanuel, T. M., Greenwood, D. R., and MacDiarmid, R. M. (2012). A critical review of translation initiation factor eIF2α kinases in plants–regulating protein synthesis during stress. Funct. Plant Biol. 39, 717-735. doi: 10.1071/FP12116

Jin, H., Axtell, M. J., Dahlbeck, D., Ekwenna, O., Zhang, S., Staskawicz, B., et al. (2002). NPK1, an MEKK1-like mitogen-activated protein kinase kinase kinase, regulates innate immunity and development in plants. Dev. Cell 3, 291-297. doi: 10.1016/S1534-5807(02)00205-8

Joubès, J., Chevalier, C., Dudits, D., Heberle-Bors, E., Inzé, D., Umeda, M., et al. (2000). "CDK-related protein kinases in plants," in Plant Cell Cycle, ed. D. Inzé (Dordrecht, Netherlands: Springer), 63-76.

Jurca, M. E., Bottka, S., and Fehér, A. (2008). Characterization of a family of *Arabidopsis* receptor-like cytoplasmic kinases (RLCK class VI). Plant Cell Rep. 27, 739-748. doi: 10.1007/s00299-007-0494-5

Kasirajan, L., Hoang, N. V., Furtado, A., Botha, F. C., and Henry, R. J. (2018). Transcriptome analysis highlights key differentially expressed genes involved in cellulose and lignin biosynthesis of sugarcane genotypes varying in fiber content. Sci. Rep. 8, 11612. doi: 10.1038/s41598-018-30033-4

Kawahigashi, H., Kasuga, S., Ando, T., Kanamori, H., Wu, J., Yonemaru, J. I., et al. (2011). Positional cloning of ds1, the target leaf spot resistance gene against *Bipolaris sorghicola* in sorghum. Theor. Appl. Genet. 123, 131-142. doi: 10.1007/s00122-011-1572-1

Khew, C. Y., Teo, C. J., Chan, W. S., Wong, H. L., Namasivayam, P., and Ho, C. L. (2015). Brassinosteroid insensitive 1-associated kinase 1 (OsI-BAK1) is associated with grain filling and leaf development in rice. J. Plant Physiol. 182, 23-32. doi: 10.1016/j.jplph.2015.05.003

Komis, G., Šamajová, O., Ovečka, M., and Šamaj, J. (2018). Cell and developmental biology of plant mitogen-activated protein kinases. Annu. Rev. Plant Biol. 69, 237-265. doi: 10.1146/annurev-arplant-042817-040314

Koo, S. C., Yoon, H. W., Kim, C. Y., Moon, B. C., Cheong, Y. H., Han, H. J., et al. (2007). Alternative splicing of the OsBWMK1 gene generates three transcript variants showing differential subcellular localizations. Biochem. Biophys. Res. Commun. 360, 188-193. doi: 10.1016/j.bbrc.2007.06.052

Krishnamoorthy, J., Mounir, Z., Raven, J., and Koromilas, A. (2008). The eIF2α kinases inhibit vesicular stomatitis virus replication independently of eIF2 phosphorylation. Cell Cycle 7, 2346-2351. doi: 10.4161/cc.6323

Lachaud, C., Prigent, E., Thuleau, P., Grat, S., Da Silva, D., Briere, C., et al. (2013). 14-3-3-regulated Ca 2+-dependent protein kinase CPK3 is required for sphingolipid-induced cell death in *Arabidopsis*. Cell Death Differ. 20, 209-217. doi: 10.1038/cdd.2012.114

Lafleur, E., Kapfer, C., Joly, V., Liu, Y., Tebbji, F., Daigle, C., et al. (2015). The FRK1 mitogen-activated protein kinase kinase kinase (MAPKKK) from *Solanum chacoense* is involved in embryo sac and pollen development. J. Exp. Bot. 66, 1833-1843. doi: 10.1093/jxb/eru524

Lee, Y., Kim, Y. J., Kim, M. H., and Kwak, J. M. (2016). MAPK cascades in guard cell signal transduction. Front. Plant Sci. 7, 80. doi: 10.3389/fpls.2016.00080

Supplementary Material

Lehti-Shiu, M. D., and Shiu, S. H. (2012). Diversity, classification and function of the plant protein kinase superfamily. Philos. Trans. R. Soc. B: Biol. Sci. 367, 2619-2639. doi: 10.1098/rstb.2012.0003

Li, J., Li, Y., Deng, Y., Chen, P., Feng, F., Chen, W., et al. (2018). A calcium-dependent protein kinase, ZmCPK32, specifically expressed in maize pollen to regulate pollen tube growth. PLoS One 13, e0195787. doi: 10.1371/journal.pone.0195787

Li, J., Wang, X. Q., Watson, M. B., and Assmann, S. M. (2000). Regulation of abscisic acid-induced stomatal closure and anion channels by guard cell AAPK kinase. Science 287, 300-303. doi: 10.1126/science.287.5451.300

Li, P., Chai, Z., Lin, P., Huang, C., Huang, G., Xu, L., et al. (2020). Genome-wide analysis of AP2/ERF transcription factors in sugarcane *Saccharum spontaneum* L. reveals functional divergence during drought, salt stress and plant hormones treatment. Res. Sq. doi: 10.21203/rs.3.rs-19836/v1

Lin, W. Y., Matsuoka, D., Sasayama, D., and Nanmori, T. (2010). A splice variant of *Arabidopsis* mitogen-activated protein kinase and its regulatory function in the MKK6–MPK13 pathway. Plant Sci. 178, 245-250. doi: 10.1016/j.plantsci.2010.01.006

Liu, J., Chen, N., Grant, J. N., Cheng, Z. M., Stewart Jr, C. N., and Hewezi, T. (2015). Soybean kinome: functional classification and gene expression patterns. J. Exp. Bot. 66, 1919-1934. doi: 10.1093/jxb/eru537

Liu, J. X., Srivastava, R., Che, P., and Howell, S. H. (2007a). Salt stress responses in *Arabidopsis* utilize a signal transduction pathway related to endoplasmic reticulum stress signaling. Plant J. 51, 897-909. doi: 10.1111/j.1365-313X.2007.03195.x

Liu, Y., Ren, D., Pike, S., Pallardy, S., Gassmann, W., and Zhang, S. (2007b). Chloroplast-generated reactive oxygen species are involved in hypersensitive response-like cell death mediated by a mitogen-activated protein kinase cascade. Plant J. 51, 941-954. doi: 10.1111/j.1365-313X.2007.03191.x

Ma, P., Yuan, Y., Shen, Q., Jiang, Q., Hua, X., Zhang, Q., et al. (2019). Evolution and expression analysis of starch synthase gene families in *Saccharum spontaneum*. Trop. Plant Biol. 12, 158-173. doi: 10.1007/s12042-019-09225-3

Malumbres, M. (2014). Cyclin-dependent kinases. Genome Biol. 15, 1-10. doi: 10.1186/gb4184

Malviya, N., Jaiswal, P., and Yadav, D. (2016). Genome-wide characterization of nuclear factor Y (NF-Y) gene family of Sorghum [*Sorghum bicolor* (L.) Moench]: a bioinformatics approach. Physiol. Mol. Biol. Plants 22, 33-49. doi: 10.1007/s12298-016-0349-z

Matschi, S., Werner, S., Schulze, W. X., Legen, J., Hilger, H. H., and Romeis, T. (2013). Function of calcium-dependent protein kinase CPK 28 of *Arabidopsis thaliana* in plant stem elongation and vascular development. Plant J. 73, 883-896. doi: 10.1111/tpj.12090

Mittal, S., Mallikarjuna, M. G., Rao, A. R., Jain, P. A., Dash, P. K., and Thirunavukkarasu, N. (2017). Comparative analysis of CDPK family in maize, *Arabidopsis*, rice, and sorghum revealed

potential targets for drought tolerance improvement. Front. Chem. 5, 115. doi: 10.3389/fchem.2017.00115

Mustilli, A. C., Merlot, S., Vavasseur, A., Fenzi, F., and Giraudat, J. (2002). Arabidopsis OST1 protein kinase mediates the regulation of stomatal aperture by abscisic acid and acts upstream of reactive oxygen species production. Plant Cell 14, 3089-3099. doi: 10.1105/tpc.007906

Roe, J. L., Rivin, C. J., Sessions, R. A., Feldmann, K. A., and Zambryski, P. C. (1993). The Tousled gene in *A. thaliana* encodes a protein kinase homolog that is required for leaf and flower development. Cell 75, 939-950. doi: 10.1016/0092-8674(93)90537-Z

Rostoks, N., Steffenson, B. J., and Kleinhofs, A. (2004). Structure and expression of the barley stem rust resistance gene Rpg1 messenger RNA. Physiol. Mol. Plant Pathol. 64, 91-101. doi: 10.1016/j.pmpp.2004.05.006

Sforça, D. A., Vautrin, S., Cardoso-Silva, C. B., Mancini, M. C., Romero-da Cruz, M. V., Pereira, G. d. S., et al. (2019). Gene duplication in the sugarcane genome: a case study of allele interactions and evolutionary patterns in two genic regions. Front. Plant Sci. 10, 553. doi: 10.3389/fpls.2019.00553

Shang, X., Cao, Y., and Ma, L. (2017). Alternative splicing in plant genes: a means of regulating the environmental fitness of plants. Int. J. Mol. Sci. 18, 432. doi: 10.3390/ijms18020432

Shi, Y., Xu, H., Shen, Q., Lin, J., Wang, Y., Hua, X., et al. (2019). Comparative analysis of SUS gene family between *Saccharum officinarum* and *Saccharum spontaneum*. Trop. Plant Biol. 12, 174-185. doi: 10.1007/s12042-019-09230-6

Sperschneider, J., Catanzariti, A.-M., DeBoer, K., Petre, B., Gardiner, D. M., Singh, K. B., et al. (2017). LOCALIZER: subcellular localization prediction of both plant and effector proteins in the plant cell. Sci. Rep. 7, 1-14. doi: 10.1038/srep44598

Stein, J. C., Howlett, B., Boyes, D. C., Nasrallah, M. E., and Nasrallah, J. B. (1991). Molecular cloning of a putative receptor protein kinase gene encoded at the self-incompatibility locus of *Brassica oleracea*. Proc. Natl. Acad. Sci. U. S. A. 88, 8816-8820. doi: 10.1073/pnas.88.19.8816

Tamaskovic, R., Bichsel, S. J., and Hemmings, B. A. (2003). NDR family of AGC kinases–essential regulators of the cell cycle and morphogenesis. FEBS Lett. 546, 73-80. doi: 10.1016/S0014-5793(03)00474-5

Tang, D., Christiansen, K. M., and Innes, R. W. (2005). Regulation of plant disease resistance, stress responses, cell death, and ethylene signaling in *Arabidopsis* by the EDR1 protein kinase. Plant Physiol. 138, 1018-1026. doi: 10.1104/pp.105.060400

Tena, G., Boudsocq, M., and Sheen, J. (2011). Protein kinase signaling networks in plant innate immunity. Curr. Opin. Plant Biol. 14, 519-529. doi: 10.1016/j.pbi.2011.05.006

Urano, F., Bertolotti, A., and Ron, D. (2000). IRE1 and efferent signaling from the endoplasmic reticulum. J. Cell Sci. 113, 3697-3702.

Supplementary Material

Vaattovaara, A., Brandt, B., Rajaraman, S., Safronov, O., Veidenberg, A., Luklová, M., et al. (2019). Mechanistic insights into the evolution of DUF26-containing proteins in land plants. Commun. Biol. 2, 1-18. doi: 10.1038/s42003-019-0306-9

Vicentini, R., de Maria Felix, J., Dornelas, M. C., and Menossi, M. (2009). Characterization of a sugarcane (*Saccharum* spp.) gene homolog to the brassinosteroid insensitive1-associated receptor kinase 1 that is associated to sugar content. Plant Cell Rep. 28, 481-491. doi: 10.1007/s00299-008-0656-0

Vikal, Y., Kaur, A., Jindal, J., Kaur, K., Pathak, D., Garg, T., et al. (2020). Identification of genomic regions associated with shoot fly resistance in maize and their syntenic relationships in the *Sorghum* genome. PLoS One 15, e0234335. doi: 10.1371/journal.pone.0234335

Vinagre, F., Vargas, C., Schwarcz, K., Cavalcante, J., Nogueira, E., Baldani, J., et al. (2006). SHR5: a novel plant receptor kinase involved in plant–N2-fixing endophytic bacteria association. J. Exp. Bot. 57, 559-569. doi: 10.1093/jxb/erj041

Wagner, T. A., and Kohorn, B. D. (2001). Wall-associated kinases are expressed throughout plant development and are required for cell expansion. Plant Cell 13, 303-318. doi: 10.1105/tpc.13.2.303

Wang, J., Li, Y., Zhu, F., Ming, R., and Chen, L. Q. (2019a). Genome-wide analysis of nitrate transporter (nrt/npf) family in sugarcane *Saccharum spontaneum* L. Trop. Plant Biol. 12, 133-149. doi: 10.1007/s12042-019-09220-8

Wang, Y., Hua, X., Xu, J., Chen, Z., Fan, T., Zeng, Z., et al. (2019b). Comparative genomics revealed the gene evolution and functional divergence of magnesium transporter families in *Saccharum*. BMC Genom. 20, 83. doi: 10.1186/s12864-019-5437-3

Wei, K., Wang, Y., and Xie, D. (2014). Identification and expression profile analysis of the protein kinase gene superfamily in maize development. Mol. Breed. 33, 155-172. doi: 10.1007/s11032-013-9941-x

Wrzaczek, M., Rozhon, W., and Jonak, C. (2007). A proteasome-regulated glycogen synthase kinase-3 modulates disease response in plants. J. Biol. Chem. 282, 5249-5255. doi: 10.1074/jbc.M610135200

Wrzaczek, M., Vainonen, J. P., Stael, S., Tsiatsiani, L., Help-Rinta-Rahko, H., Gauthier, A., et al. (2014). GRIM REAPER peptide binds to receptor kinase PRK 5 to trigger cell death in *Arabidopsis*. EMBO J. 34, 55-66. doi: 10.15252/embj.201488582

Xu, N., Gao, X. Q., Zhao, X. Y., Zhu, D. Z., Zhou, L. Z., and Zhang, X. S. (2011). *Arabidopsis* AtVPS15 is essential for pollen development and germination through modulating phosphatidylinositol 3-phosphate formation. Plant Mol. Biol. 77, 251. doi: 10.1007/s11103-011-9806-9

Xu, S., Wang, J., Shang, H., Huang, Y., Yao, W., Chen, B., et al. (2018). Transcriptomic characterization and potential marker development of contrasting sugarcane cultivars. Sci. Rep. 8, 1-11. doi: 10.1038/s41598-018-19832-x

Yadeta, K. A., Elmore, J. M., Creer, A. Y., Feng, B., Franco, J. Y., Rufian, J. S., et al. (2016). A cysteine-rich protein kinase associates with a membrane immune complex and the cysteine residues are required for cell death. Plant Physiol. 173, 771-787. doi: 10.1104/pp.16.01404

Yan, J., Li, G., Guo, X., Li, Y., and Cao, X. (2018). Genome-wide classification, evolutionary analysis and gene expression patterns of the kinome in *Gossypium*. PLoS One 13, e0197392. doi: 10.1371/journal.pone.0197392

Yan, J., Su, P., Wei, Z., Nevo, E., and Kong, L. (2017). Genome-wide identification, classification, evolutionary analysis and gene expression patterns of the protein kinase gene family in wheat and *Aegilops tauschii*. Plant Mol. Biol. 95, 227-242. doi: 10.1007/s11103-017-0637-1

Yang, R., Wek, S. A., and Wek, R. C. (2000). Glucose limitation induces GCN4Translation by activation of Gcn2 protein kinase. Mol. Cell. Biol. 20, 2706-2717. doi: 10.1128/MCB.20.8.2706-2717.2000

Yang, X., Sood, S., Luo, Z., Todd, J., and Wang, J. (2019). Genome-wide association studies identified resistance Loci to orange rust and yellow leaf virus diseases in sugarcane (*Saccharum* spp.). Phytopathology 109, 623-631. doi: 10.1094/PHYTO-08-18-0282-R

Yu, C. S., Chen, Y. C., Lu, C. H., and Hwang, J. K. (2006). Prediction of protein subcellular localization. PROTEINS: Struct. Funct. Bioinform. 64, 643-651. doi: 10.1002/prot.21018

Zhang, Y., Shewry, P. R., Jones, H., Barcelo, P., Lazzeri, P. A., and Halford, N. G. (2001). Expression of antisense SnRK1 protein kinase sequence causes abnormal pollen development and male sterility in transgenic barley. Plant J. 28, 431-441. doi: 10.1046/j.1365-313X.2001.01167.x

Zhang, Z., Li, J., Zhao, X. Q., Wang, J., Wong, G. K. S., and Yu, J. (2006). KaKs_calculator: calculating Ka and Ks through model selection and model averaging. Genom. Proteom. Bioinform. 4, 259-263. doi: 10.1016/S1672-0229(07)60007-2

Zhu, K., Liu, H., Chen, X., Cheng, Q., and Cheng, Z. M. M. (2018a). The kinome of pineapple: catalog and insights into functions in crassulacean acid metabolism plants. BMC Plant Biol. 18, 1-16. doi: 10.1186/s12870-018-1389-z

Zhu, K., Wang, X., Liu, J., Tang, J., Cheng, Q., Chen, J. G., et al. (2018b). The grapevine kinome: annotation, classification and expression patterns in developmental processes and stress responses. Hortic. Res. 5, 1-16. doi: 10.1038/s41438-018-0027-0

Zulawski, M., Schulze, G., Braginets, R., Hartmann, S., and Schulze, W. X. (2014). The *Arabidopsis* kinome: phylogeny and evolutionary insights into functional diversification. BMC Genom. 15, 1-15. doi: 10.1186/1471-2164-15-548
